# The Development of Third-Generation Tetracycline Antibiotics and New Perspectives

**DOI:** 10.3390/pharmaceutics13122085

**Published:** 2021-12-05

**Authors:** Aura Rusu, Emanuela Lorena Buta

**Affiliations:** Pharmaceutical and Therapeutical Chemistry Department, Faculty of Pharmacy, George Emil Palade University of Medicine, Pharmacy, Science and Technology of Targu Mures, 540142 Targu Mures, Romania; lorrenush@yahoo.com

**Keywords:** tetracyclines, structure-activity relationship, mechanism, antibacterial activity, resistance, fluorocycline, aminomethylcycline, glycylcycline

## Abstract

The tetracycline antibiotic class has acquired new valuable members due to the optimisation of the chemical structure. The first modern tetracycline introduced into therapy was tigecycline, followed by omadacycline, eravacycline, and sarecycline (the third generation). Structural and physicochemical key elements which led to the discovery of modern tetracyclines are approached. Thus, several chemical subgroups are distinguished, such as glycylcyclines, aminomethylcyclines, and fluorocyclines, which have excellent development potential. The antibacterial spectrum comprises several resistant bacteria, including those resistant to old tetracyclines. Sarecycline, a narrow-spectrum tetracycline, is notable for being very effective against *Cutinebacterium acnes*. The mechanism of antibacterial action from the perspective of the new compound is approached. Several severe bacterial infections are treated with tigecycline, omadacycline, and eravacycline (with parenteral or oral formulations). In addition, sarecycline is very useful in treating acne vulgaris. Tetracyclines also have other non-antibiotic properties that require in-depth studies, such as the anti-inflammatory effect effect of sarecycline. The main side effects of modern tetracyclines are described in accordance with published clinical studies. Undoubtedly, this class of antibiotics continues to arouse the interest of researchers. As a result, new derivatives are developed and studied primarily for the antibiotic effect and other biological effects.

## 1. Introduction

**Figure 1:**
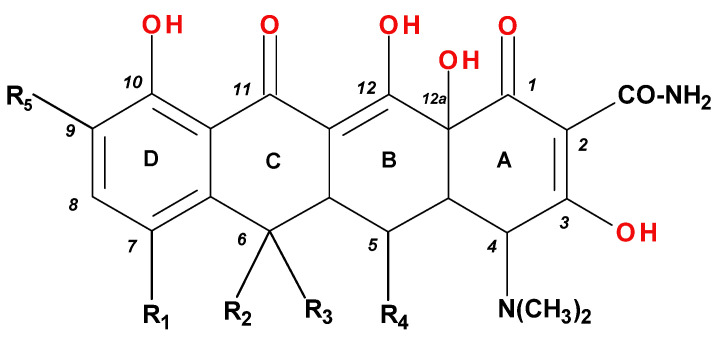
Tetracyclines—the general chemical structure and conventional numbering of the condensed rings and key positions.

Tetracyclines are an important class of broad-spectrum antibiotics that prevent bacterial growth by inhibiting protein biosynthesis. This large family includes compounds with bacteriostatic activity and a wide range of uses, from Gram-positive and Gram-negative bacterial infections to those caused by a protozoan parasite and intracellular organisms [1]. Sarecycline is unique, being the only narrow-spectrum antibiotic in the tetracycline-class family. The basic structure of tetracyclines consists of four linearly condensed benzene rings in a hydronaphtacene nucleus. The essential differences between the analogues of this class are given by the C5, C6, C7, and C9 substituents (Figure 1) [2].

### 1.1. Brief History of Tetracycline Antibiotics

The emergence of tetracycline development is due to the contribution of hundreds of dedicated researchers, scientists, and clinicians over more than 60 years [3]. Since their discovery (1948, aureomycin), tetracyclines have played an essential role in treating bacterial infections [4]. Stimulated by the extraordinary success of penicillins, several companies and academic institutions have focused on discovering new antibiotics produced by microorganisms, analysing numerous samples of soil sent from different parts of the world. It was observed that actinomycete bacteria produced a yellow colony, with a remarkable inhibitory effect against many pathogenic strains, including rickettsia and Gram-positive and Gram-negative bacteria. This actinomycete bacteria became famous for its broad-spectrum antibiotic. The first tetracycline was extracted from *Streptomyces aureofaciens* and was named aureomycin (syn. chlortetracycline) [5,6,7,8]. Professor Benjamin Minge Duggar supervised the discovery of the first tetracycline. After the Food and Drug Administration (FDA) approval in 1948, aureomycin saved many lives and brought fame and profit to the Cyanamid (Lederle Laboratories Division) manufacturing company, being successfully marketed [3,7,9,10]. After Pfizer isolated *Streptomyces rimosus*, the aureomycin and terramycin (syn. oxytetracycline) were discovered [7]. This new compound was the second representative of this class of antibiotics, similar in chemical structure but with superior bioavailability and water solubility. The FDA approved Terramycin in 1950 [3,9,11].

Tetracycline was discovered in 1953, on the basis of the chemical structure of chlortetracycline, by catalitic hidrogenation (with palladium and hydrogen). The new antibiotic agent presented and improved the pharmacokinetic profile, which quickly became a favourite in therapy [12,13]. This remarkable success has proven for the first time in history that other biologically active and valuable antibiotics can be obtained by operating changes on the basic structure (molecule optimisations) [3,14]. After discovering chlortetracycline, oxytetracycline, and tetracycline (first generation of tetracyclines), the chemists of Pfizer and Lederle Laboratories began the development of new tetracyclines, with superior pharmacokinetic properties, wider antimicrobial spectrum, and lower toxicity [15]. Among the discovered representatives were methacycline (1966), doxycycline (1967), and minocycline (1972) [13,16]. Doxycycline is a semisynthetic analogue based on the chemical structure of the metacycline, approved in 1967 by the FDA. These tetracyclines are classified in the second generation (Table S1—Supplementary Materials) [3,14,17].

The further development of semisynthetic analogues of the second generation, and, more recently, of the third generation (Table 1), reveals the evolution of this class. The modern tetracyclines had acquired high potency and an increased efficacy, even against bacteria resistant to tetracyclines [18,19,20]. Therefore, biochemical mutants of *Streptomycetes* strains have been created for a higher production yield and to discover novel tetracyclines [3,15].

Therefore, compounds such as demeclocycline were discovered, being later converted in 1971 to a C7-amino-derivative known as minocycline. Some authors classified minocycline as the last tetracycline in the second generation. Moreover, demeclocycline was a precursor of sancycline (obtained by reduction), a tetracycline with a simplified chemical structure and retained biological activity [1,25]. Thus, the main advantage of minocycline is a broader spectrum of activity compared to previously representatives. Furthermore, minocycline presented a better pharmacokinetic profile and was the most potent representative at the time, being the last introduced on the market in the next three decades [3,17,26].

### 1.2. The Discovery of Modern Tetracyclines

**Figure 2:**
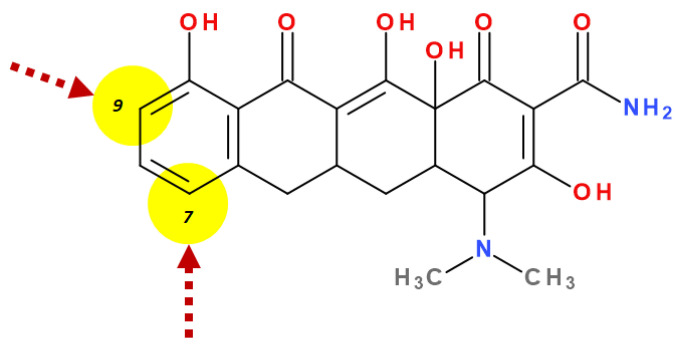
The chemical structures of sancycline; key positions highlighted C7 and C9 for structural design optimisation to obtain new derivatives.

The growing occurrence of bacterial resistance to antibiotics has once again aroused the interest of scientists in the development of new tetracyclines. Thus, at the end of the 1980s, the programs were reopened for the synthesis of new compounds that could be classified into a new generation (the third one), re-evaluating the compounds already synthesised (Table S2—Supplementary Materials) [3]. The main interest was about the modification of C7 and C9 positions of the D ring in the sancycline structure (Figure 2, Table S3—Supplementary Materials). These steps have led to the discovery of a novel class of C9-aminotetracyclines, which bear a glycyl moiety known as *glycylcycline* [3,27,28,29].

**Figure 3:**
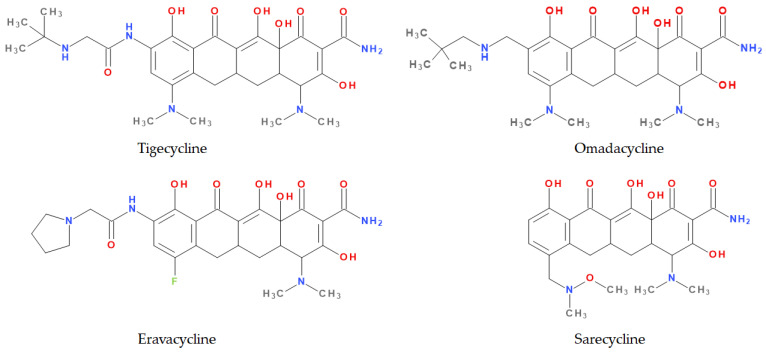
The chemical structure of the representatives of the third-generation tetracyclines.

Modern tetracyclines include derivatives with more or less similar chemical structures: a glycylglicine (tigecycline), an aminomethylcycline (omadacycline), a fluorocycline (eravacycline), and a 7-[(methoxy-(methyl)-amino)-methyl]methyl] derivative (sarecycline) (Figure 3).

Tigecycline is a synthetic derivative of minocycline discovered in 1993. Tigecycline was the first tetracycline introduced in therapy after more than 30 years. Thus, tigecycline could be considered the prototype of a new subclass of tetracyclines [27,29]. This new tetracycline has the advantage of a superior potency over Gram-positive and Gram-negative multidrug-resistant bacteria (multiple drug resistance, MDR) [26,30]. Tigecycline was discovered by Wyeth Pharmaceuticals Inc. and approved by the FDA in 2005 [31] and later by the European Medicine Agency (EMA) in 2006, under the trade name Tygacil [32]. Tygacil received approval for complicated intra-abdominal and complicated skin and soft tissue infections [33,34]. Likewise, in 2008, the FDA approved the use of tigecycline to treat community-acquired pneumonia [35,36]. Once placed in the market, several other uses have been investigated: nosocomial pneumonia, diabetic foot infections, emergency therapy for MDR pathogens, nosocomial urinary tract infections, and *Clostridium difficile* infections [26]. A disadvantage of tigecycline is its exclusive parenteral use due to its poor bioavailability [37].

Omadacycline is one of the newest and most popular tetracyclines and the first in the aminomethylcycline subclass [3,38]. It has a broad spectrum of activity, proving in vitro effects against Gram-positive and Gram-negative bacteria, anaerobic bacteria, and atypical bacteria. In addition, the activity of this compound extends to methicillin-resistant *Staphylococcus aureus* (MRSA); penicillin-resistant, MDR *Streptococcus pneumoniae*; and vancomycin-resistant enterococci [22]. Unlike tigecycline, omadacycline is available for both oral and parenteral administration. Both forms were approved in 2018 by the FDA for the treatment of complicated intra-abdominal and complicated skin and soft tissue infections and community-acquired pneumonia. Currently, omadacycline is in phase II of clinical trials to treat urinary tract infections, such as acute pyelonephritis and cystitis [38]. The pharmaceutical product Nuzyra was approved in the United States of America (USA) [39].

Eravacycline is a synthetic fluorocycline, obtained by total synthesis, that contains a basic chemical structure of the tetracyclines class [40,41]. In addition, particular modifications on the D ring of the naphtacen nucleus were introduced. Those chemical optimisations give it a remarkable activity against Gram-positive and Gram-negative bacteria that developed specific resistance mechanisms to the tetracycline antibiotic class to treat complicated intra-abdominal infections in adults. It is available for parenteral administration in many countries in Europe, as well as in the USA [41].

Sarecycline is an analogue of tetracycline specifically designed for the treatment of acne [42]. It is available as an oral formulation to treat inflammatory lesions of moderate to severe non-nodular acne vulgaris. The main advantage of this new tetracycline is a higher selective activity against *Cutinebacterium acnes* comparative to older tetracyclines (doxycycline and minocycline) used in acne therapy. Due to this selectivity, the probability of developing antibiotic resistance is lower than minocycline and doxycycline [43]. Sarecycline was developed by Paratek Pharmaceuticals and Allergan but acquired by Almirall S.A by purchasing the dermatological portfolio [44]. The FDA approved sarecycline in 2018 under the trade name Seysara [45,46].

## 2. Research Methodology

The relevant primary data were found on Clarivate Analytics and ScienceDirect databases using the following keywords: (i) topic: “tetracyclines”, “antibacterials”; (ii) title: “tigecycline”, “omadacycline”, “eravacycline”, “sarecycline”, and other classic derivatives of the tetracycline class. In the second stage, the articles were selected if they comprised development of tetracyclines class, physicochemical properties, aspects related to structure–activity relationships, new tetracyclines design, mechanism of action, antibacterial spectrum, therapeutical value, safety profile, bacterial resistance, and new derivatives in development. The paper includes significant references, including the latest articles published in 2021.

All chemical structures were drowned with Biovia Draw 2019 (San Diego, CA, USA) [47].

## 3. Overview of Modern Tetracyclines

This paper’s primary targeted the new tetracyclines classified as the third generation: tigecycline, omadacycline, eravacycline, and sarecycline.

### 3.1. Considerations Regarding the Chemical Structure and Physicochemical Properties of the New Tetracyclines

Recently introduced compounds in the tetracycline class contain the basic chemical structure specific to this class, four condensed rings (A, B, C, and D) into a naphtacen-carboxamide system. Other common structural elements are a dimethyl-amino group at the C4 position, an amidic group at the C2 position, a keto–enol alternation system (C11, C12, and 12a positions), and asymmetric carbons at the junction of rings A-B (stereochemical configurations) (Figure 1 and Figure 3) [21,48,49]. X-ray crystallography of tetracycline, doxycycline, and sancycline revealed that the C2 amide group is oriented to form an intramolecular hydrogen bond with oxygen atom from C3 position [50]. The above elements are considered the minimum pharmacophore (6-deoxy-dimethyltetracycline) required for antimicrobial activity and a start point for inserting other substituents [21,48,49].

Depending on the radicals grafted on the tetracyclic system, these new molecules introduced on the market after 2000 present different physicochemical and pharmacological characteristics and changes in the antimicrobial spectrum [13,21,38,41,44]. The optimisation of the basic structure consisted of C7 and C9 substitutions. Position C7 is subject to substitution with electron acceptor or donor groups [40]. Thus, tigecycline and omadacycline have a dimethyl-amino group in this position. Eravacycline has a fluorine atom at the C7 position, being an electron-withdrawing substituent [40,51]. In the same place, sarecycline has a more voluminous radical, methoxy-methyl-amino-methyl [42]. The radicals in the C9 position are distinct for each of the new representatives. Tigecycline and eravacycline are synthetic analogues that contain a glycyl-amide substituent [40,52].

**Figure 4:**
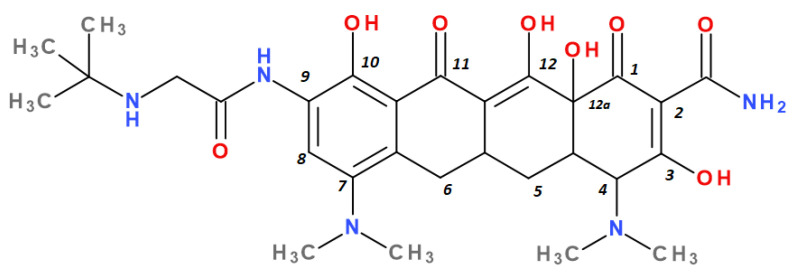
The chemical structures of the tigecycline and conventional numbering.

Tigecycline was synthesised by adding a *tert*-butyl-glycyl-amide substituent, while in eravacycline this group was replaced with a pyrrolidinyl-acetamide group [32,40]. However, tigecycline is an analogue of minocycline formed by adding the *tert*-butyl-glycyl-amide substituent at the C9 position (Figure 4). It is the first glycylcycline tetracycline discovered [29,31]. Currently, tigecycline is manufactured as a lyophilised powder form because it undergoes a degradation process. Tigecycline is commercialised under the trade name Tygacil (pharmaceutical form for intravenous infusion). The recommended doses regimen is 100 mg initial dose, followed by 50 mg every 12 h [31].

**Figure 5:**
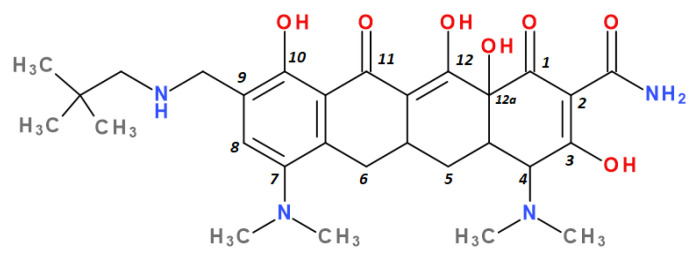
The chemical structures of the omadacycline and conventional numbering.

Omadacycline (sin. amadacycline) is the first aminomethylcycline of this new subclass, for which the glycyl-amide group was changed to an alkyl-amino-methyl group [38,53,54]. The optimisation at the C9 atom was based on a methyl(2,2-dimethylpropylamino) fragment that replaces the glycylamide group present in the case of its homologues (tigecycline and eravacycline) (Figure 5) [52]. Omadacycline is formulated as tosylate salt for intravenous or oral administration under the trade name Nuzyra [38].

**Figure 6:**
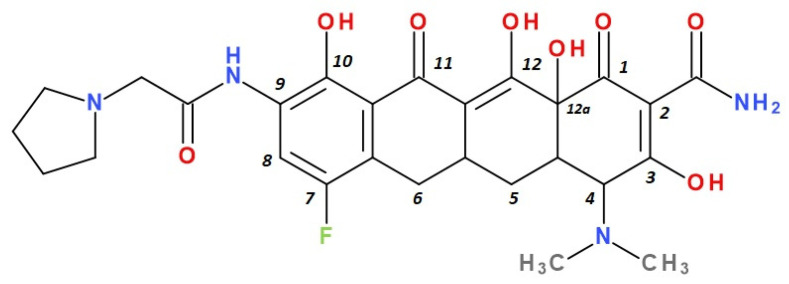
The chemical structures of the eravacycline and conventional numbering.

The primary structure of tetracyclines is maintained in the chemical structure of eravacycline, which is an analogue of tigecycline, with two changes on the D ring: the addition of a fluorine atom in the C7 position and the substitution of the *tert*-butyl-amino-acetamide group in the C9 position with a pyrrolidin-acetamido group (Figure 6) [41]. Pharmaceutical formulation under the trade name Xerava contains eravacycline, powder for concentrate for solution for infusion (50 mg and 100 mg) for intravenous use [55].

**Figure 7:**
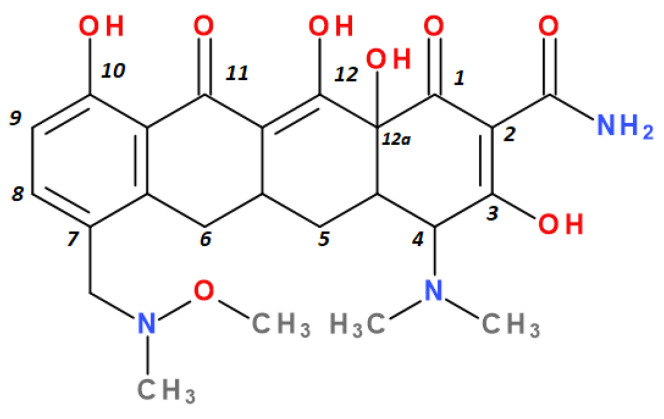
The chemical structures of the sarecycline and conventional numbering.

Sarecycline has no substitute in the C9 position [56]. Sarecycline is chemically distinguishable from other tetracycline-class antibiotics by the 7 [[methoxy(methyl)amino]methyl] group attached at the C7 position of the ring D. This stable modification represents the longest and the largest C7 moiety among all of the tetracyclines (Figure 7). Sarecycline inhibits bacterial ribosomes through interactions with the mRNA as a consequence of C7 optimisation. This new tetracycline blocks accommodation into the A site of the first aminoacyl transfer RNA and appears to be a more potent initiation inhibitor comparative to previous analogues [57]. Sarecycline is manufactured as hydrochloride salt [42]. Pharmaceutical formula Seysara tablets for oral use contains sarecycline (60 mg, 100 mg, 150 mg) [46].

Essential physicochemical properties of the third generation tetracyclines are shown in Table S4 (Supplementary Materials).

**Figure 8:**
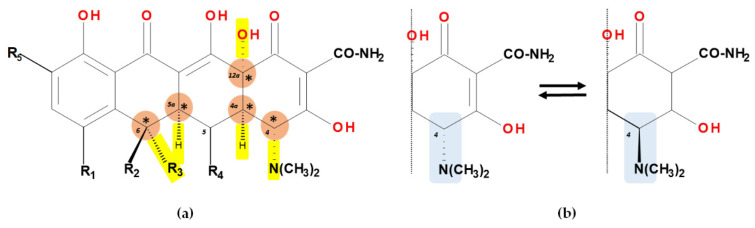
The chiral atoms on the chemical structure of tetracyclines and conventional numbering (**a**); epimerisation of tetracyclines (**b**); *—chiral centers.

Tetracyclines are optically active substances. The X-ray diffraction analysis (XRD) established the stereochemistry of the basic structure of these compounds. Depending on substitution, several chiral atoms are C4, C4*a*, C5, C5*a*, C6, and C12*a* (Figure 8a). Some derivatives, such as oxytetracycline and doxycycline, have six chiral carbon atoms, due to the C5α-hydroxyl substituent. Moreover, a conjugated system is known in the naphtacene nucleus (C10 to C12; C1 to C3) [58].

In acidic conditions, tetracyclines epimerase reversibly at the C4 position (Figure 8b). The resulted isomers are known as “epitetracyclines”, founded in equal amounts after establishing the equilibrium. The formation of 4-epitetracyclines is notable because they are less active than non-epimerised isomers [58,59].

Tetracyclines are amphoteric compounds due to the characteristic structural elements (hydroxyls and dimethylamino substituents and the conjugated keto-enolic system). In reaction with an acid or a base, tetracyclines form salts. In pharmaceutical formulations, tetracyclines are most commonly used in the form of hydrochloric salts (e.g., eravacycline, sarecycline). Depending on the solvent, the tetracyclines’ structure changes from an ionised to a non-ionised state (protonation–deprotonation equilibria). At the neutral pH, tetracyclines mainly adopt the zwitterion form. It is known that acid salts of tetracyclines exhibit a minimum of three acidity constants in aqueous solutions [14,50,59,60].

**Figure 9:**
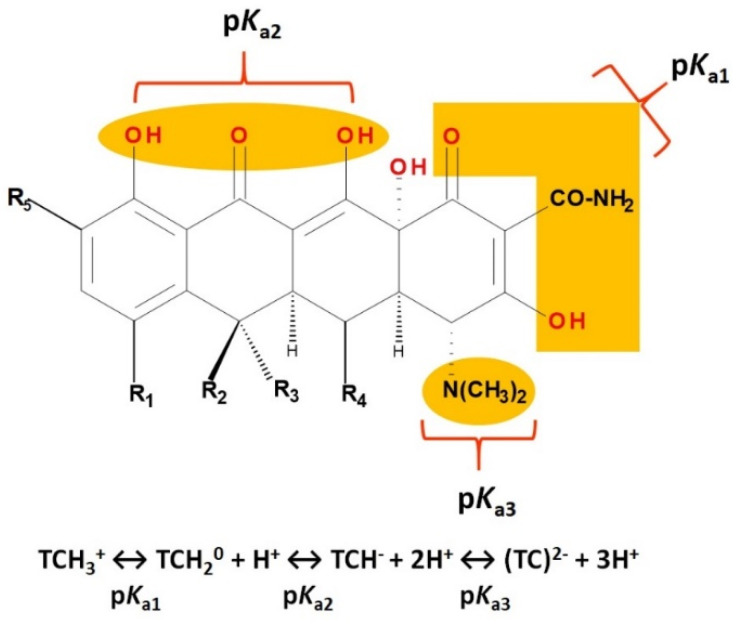
The tetracycline (TC) structural sites and the correspondent acidic dissociation constants [61,62,63,64,65].

The main protonation sites of tetracyclines are the tricarbonyl system (C1-C2-C3), phenolic diketone-system (C10-C11-C12), and dimethylamino group (C4) (Figure 9) [58,61,62,63]. Depending on the substituents on the basic chemical structure, the protonation state of the compound also changes [64]. Tetracyclines are multiprotic compounds. Put simply, tetracyclines can be considered to behave similar to triprotic acids [63]. Other authors have suggested that tetracyclines have four ionisation equilibria and four correspondent pKa values (at pH values of 3.2, 7.6, 9.6, and 12) and five protonation states [63].

Thus, tigecycline poses five main ionisation groups, specifically, five values of p*K*_a_ (at pH values of 2.8, 4.4, 7.4, 8.9, and 9.5), an important role having the substitutes from C7 and C9 [65]_._ Using MarvinSketck (ChemAxon, Budapest, Hungary) for sarecycline, researchers found 17 possible microspecies depending on the pH value. Table 2 comprises the predicted microspecies and the highest value of ionisation (%) at a specific pH; additionally, the degree of ionisation at the physiologic pH (7.4) was highlighted. The highest ionisation percentages of the microspecies were identified as follows: no. 3 at pH 0–1 (98.15%), no. 6 at pH 4 (98.66%), no. 14 at pH 11 (96.30%), no. 17 at pH 14 (95.55%), and no. 7 at pH 8.6 (49.38%). At the physiologic pH (7.4), no. 2 was 21.59%, the highest ionisation percentage.

Protonation equilibria and formed microspecies play an essential role in the bioavailability of tetracyclines [63]. The protonated state of tetracyclines is also essential in the analysis. For example, in electrospray mass spectrometry, both protonated molecules of tigecycline (MH+ and MH_2_^2+^) are predominantly formed [67].

Electron-rich functional groups depending on pH can be protonated or deprotonated. Consequently, the tetracyclines have excellent chelating properties with several bivalent or trivalent metal cations [60]. Thus, tetracyclines form stable complexes with metal ions due to the characteristic substituents (Table S3—Supplementary Materials) [60].

First-generation tetracyclines form insoluble complexes with metal ions (Ca^2+^, Mg^2+^, Fe^3+^, and Al^3+^) and consequently present reduced absorption [49]. Doxycycline and minocycline (second generation) are known for their excellent ability to chelate Fe^3+^. Both doxycycline and minocycline absorption are impaired by ferrous sulphate; bismuth; and other antacids containing aluminium, calcium, and magnesium salts, such as co-administration of pharmaceuticals with Fe^3+^ and antacids (rich in Ca^2+^, Mg^2+^) [49,68].

Minocycline (second generation) and tigecycline (third generation) are more chelated by Ca^2+^ than tetracycline due to the C7 dimethylamino group. This moiety increased the electron density at the Ca^2+^ coordination site for the two studied tetracyclines. In addition, it was observed that tigecycline formed a different higher-order complex comparative to minocycline through the C9 N-t-butylglycylamido substituent in Ca^2+^ coordination [65]. Complexes with magnesium ions inhibit bacterial growth by impairing protein synthesis; these tetracycline complexes with magnesium act by binding to the 30S ribosomal subunits [69]. In plasma, tetracyclines are mainly chelated with Ca^2+^ and Mg^2+^ ions. A known mechanism of bacterial resistance to tetracyclines involves metal complexation. A possible strategy to combat bacterial resistance is to use in therapy the metal complexes of tetracycline (e.g., Pt^2+^ or Pd^2+^ complexes) [69,70].

### 3.2. Structure-Activity Relationships

As a result of studies of the relationship between chemical structure and biological activity, several aspects related to the class of tetracyclines are already known [3,13,48,49,71]. Next, the structural elements with an impact on the biological properties of the new tetracyclines are targeted.

**Figure 10:**
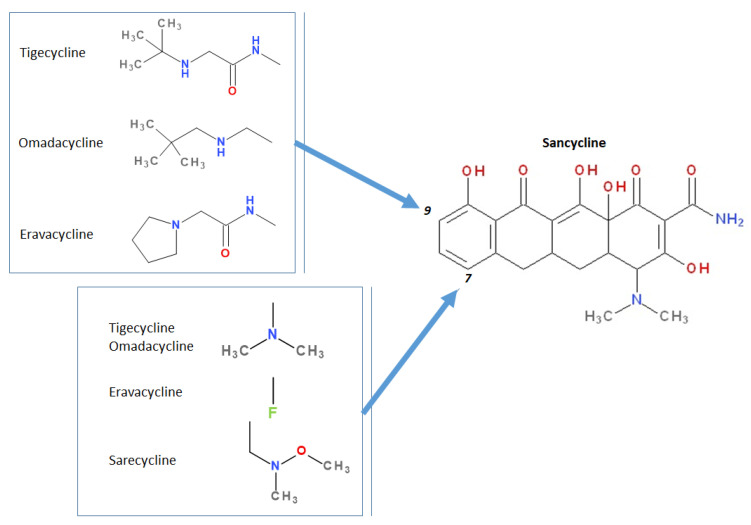
Optimisation of C7 and C9 positions in the development of new tetracyclines.

Tigecycline was discovered as a result of chemical structure–activity studies [72]. Due to structural modifications made to C9 position (Figure 10), tigecycline has an affinity for the ribosomal target five times higher than tetracycline or minocycline. Therefore, this change is responsible for broadening the antibacterial spectrum and combating ribosomal protection, one of the two mechanisms of bacterial resistance specific to tetracycline [29,73,74]. Moreover, this radical is a bulky steric hindrance that prevents the expulsion of the substance out of the bacterial cell by effluent tet proteins, thus reducing the susceptibility of developing antibiotic resistance [28,75].

Researchers observed some structural features of glycylcyclines that are essential in maintaining biological activity [13]. A critical element is the basic nitrogen atom from the glycyl unit; derivatives containing low volume alkyl-amino or cyclic amine groups have shown optimal results. Attempts to replace the radical from the C9 position with other amino acids such as alanine, phenylalanine, and leucine failed because the resulting compounds were much less effective [72]. Similarly, substitutions with smaller groups than the *tert*-butyl-amino group led to compounds with low potency, while the attempt to substitute the amine with n-propyl, n-butyl, and n-hexyl has not brought improvements [76]. The antimicrobial activity and the pharmacokinetics of tigecycline are considerably influenced by the ability to form complexes with metal ions (calcium, magnesium, and iron). The target of the cations is the keto–enol system (C11 and C12 positions), the enol in position C1, and the carboxamide in position C2. Therefore, water-insoluble chelates are formed with a low absorption [13].

Chemical modulations performed to obtain omadacycline on C9 led to an increase in antimicrobial potency by overcoming the resistance to the efflux mechanisms and overcoming ribosomal protection [22,77]. Furthermore, the aminomethyl moiety from C9 position provides improved pharmacokinetic parameters, such as dose reduction (high doses cause side effects such as nausea and vomiting, often encountered in C9 glycylcyclines), and, in particular, oral bioavailability [78]. Due to these changes, omadacycline has a pharmacokinetic profile (absorption, distribution, metabolism, and excretion, ADME) that distinguishes it from the glycylcycline subgroup [77].

A study was conducted on aminomethycyclines with in vitro potency (with a minimum inhibitory concentration (MIC) ≤ 0.06–2.0 μg/mL) against Gram-negative bacteria with different mechanisms of resistance by ribosomal protection (Tet(M)): *Staphylococcus aureus, Enterococcus faecalis,* and *Streptococcus pneumoniae,* and on the efflux mechanisms Tet(K) in *Staphylococcus aureus*, Tet(L) in *Enterococcus faecalis*) [51]. Compounds with lipophilia-enhancing or benzyl substitutions in the aminomethyl side group showed the highest potency against ribosomal alteration and efflux of resistant strains. However, high-polarity analogues or electrically charged groups, as well as acyl derivatives, showed a significant decrease in antibacterial activity. Although alkyl substituents (e.g., *tert*-butyl group) showed moderate potency, they were chosen for further optimisation and screening. It was found that analogues with the alkyl group, which extend with at least three carbon atoms to the aminomethyl group; those with branched alkyl chains; and piperidine analogues have superior activity. The ramification in the alkyl chain from position 1 has a detrimental impact due to steric hindrance. The introduction of two methyl groups in position 2 showed a significant improvement in antibacterial activity. Finally, residues containing more than five carbon atoms had reduced activity in the presence of plasma, indicating a high percentage binding to plasma proteins [38,51].

Therefore, following classical studies on the chemical structure–biological activity relationships, omadacycline, a compound containing a neopentyl moiety in the aminomethyl group, has been identified as the most valuable aminomethylcycline in this series, becoming a new subclass of tetracyclines [77].

It has been observed on fluorocyclines that as the substituents attached to the carbon atom at the C9 positions are more polar or more basic, the microbiological activity of the compound will increase, especially on Gram-negative bacteria. A study conducted in 2012 examined the behaviour of analogues of 7-fluoro-9-amino-acetamido-6-demethyl-6-deoxytetracyclines on various Gram-negative and Gram-positive bacteria, but also on gene isolates resistant to tetracycline class [79]. In general, less voluminous secondary or tertiary amine analogues from C9 position were found to have a lower MIC compared to analogues with substituents such as aromatic amines or alkylamines with lower basicity. Compared to tertiary alkylamine, dimethyl, azetidine, and piperidine analogues, eravacycline, a compound bearing a pyrrolidine nucleus, showed a 8 to 16 times higher potency against *K. pneumoniae* (tet(A)) and 4 to 8 times higher potency against *E. coli* (tet(A)). In addition, eravacycline is 4 to 64 times more potent than piperidine and azetidine omologues against bacterial isolates that have been tested (except for *S. pneumoniae* expressing or not expressing tet(M) protein, were it showing an equivalent response). The addition of polar substituents, fluorine atoms, or pyrrolidine bicycles produced no improvements, but neither did negative influence on the activity against pneumococcal bacteria when compared to unsubstitued pyrrolidine analogues [40,80]. The pyrrolidine substituent at the C9 the fluoro substituent at C7, the main optimisations in eravacycline, positively influenced the potency and the antibacterial spectrum [19].

Unlike other tetracyclines, the chemical structure of sarecycline includes a unique modification at the C7 position (the longest and most voluminous of the whole class), a 7-[(methoxy-(methyl)-amino)-methyl]methyl group (Figure 10). As a result of this chemical optimisation, the activity of this compound is enhanced, binding to the codon of the A site, interfering with the movement of messenger RNA (mRNA) along the channel, or disrupting the codon A-anticodon interaction [42].

**Figure 11:**
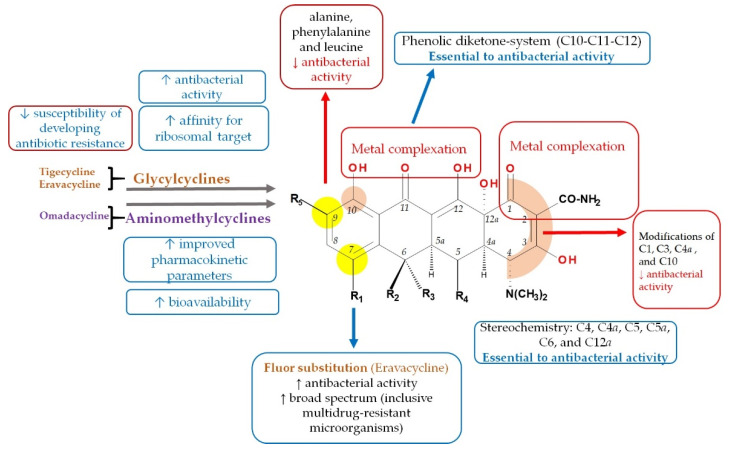
The essential relationship between chemical structure and biological activity of modern tetracyclines.

The substituted tetracycline system at positions C7 and C9 is the basis of compounds with increased antibacterial activity, while any modification made at the C1-C4, C10-C12, C-11a, and C-12a will have a negative consequence on their action. Other important aspects regarding the relationship between chemical structure and biological activity are presented in Figure 11 [3,38,40,51].

In addition, tetracyclines contain a 4S(α)-dimethyl-amino group in the C4 position, an absolute necessity for optimal antibacterial activity. On the other hand, the epimerisation of the 4R(β) isomer will lead to a decrease in antibacterial activity, especially against Gram-negative bacteria. The epimerisation process from position C4 takes place during harsh chemical reactions, in vivo metabolism phenomenon, but also under changes in the pH values [25].

The C4 β-epimers have noticeably different properties from those of compounds with a normal configuration. The most significant difference is observed in antibacterial activity manifested in vitro. β-Epimers have been found to be responsible for approximately 5% of normal tetracycline activity. It has been observed that the epimerisation phenomenon takes place in different solvent systems, at variations of pH between 2 and 6 [14]. Tetracyclines are prone to epimer formation, particularly under weak acidic conditions. The epimers have distinct toxicological and antibacterial properties, and therefore selective biosynthesis is a major challenge. This is due to the fact that the epimers are isobars with the parent compound, having very similar physico-chemical properties. Epimerisation can occur in vivo, even in the bladder [59].

### 3.3. Mechanism of Action

**Figure 12:**
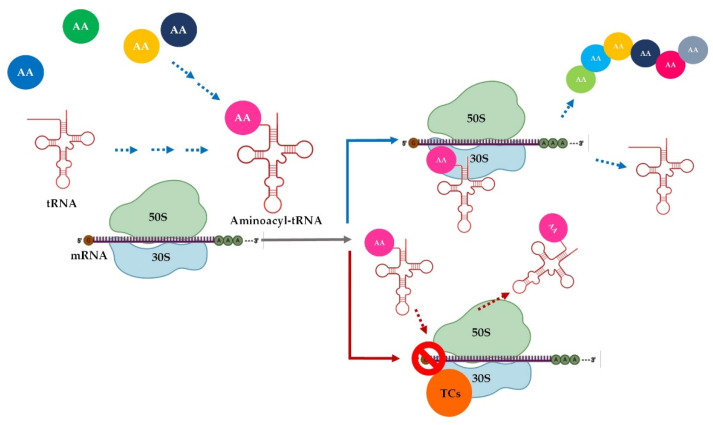
Scheme of the tetracyclines’ mechanism of action, where AA—aminoacids, TCs—tetracyclines, tRNA—transfer ribonucleic acid, mRNA—messenger ribonucleic acid, 30S and 50S—ribosomal subunits (created with BioRender.com (accessed on 30 September 2021) [84].

Tetracyclines inhibit protein synthesis by inhibiting the association of aminoacyl-tRNA with bacterial ribosome [49,75]. Tetracyclines bind with high affinity to a specific locus (16S) on the 30S ribosomal unit during translation. In this way, the penetration of aminoacyl transporter RNA (tRNA) into the acceptor site (A) on the bacterial ribosome is blocked, the consequence being the cessation in the incorporation of amino acids residues in the process of elongation of the polypeptide chain. Thus, the protein synthesis is stopped (Figure 12) [18,81,82]. Commonly, at therapeutic concentrations, tetracyclines are consider bacteriostatic antibiotics [18], but late studies have described their bactericidal effects in vitro, especially in the case of tigecycline (studies on mice) [83].

It is well known that tetracyclines cross the membranes of Gram-negative bacteria through a cationic complex with Mg^2+^, using the OmpF and OmpC porins in the outer membrane [38,85]. Later on, the Donnan potential generated along the outer membrane causes the accumulation of the complex in the periplasmic space, where the dissociation from the Mg^2+^ ion of tetracycline takes place and there is a release of an electrically uncharged molecule that is lipophilic enough to diffuse through the inner membrane into the cytoplasm [86]. The uptake of tetracyclines in the cytoplasm is partially energy-dependent, involving, in addition to passive diffusion, the proton-motive force and the hydrolysis of phosphate bonds [18]. For Gram-positive bacteria, it has been reported that these agents reach the cytoplasm by passive diffusion and/or active transport. In the cytoplasm, tetracyclines chelate Mg^2+^ ions again and, in this form, attack the ribosomal target [75]. Hence, bivalent ions are a vital element in the transport and efficiency of these compounds [85].

Tigecycline is not affected by most common antibiotic resistance mechanisms because it binds to the 30S subunit (five times stronger than tetracyclines) [28], even in the presence of ribosomal protection, being excepted from membrane efflux [87]. This is due to the voluminous substituent in the C9 position of the naphtacenic nucleus, representing a steric hindrance [88,89]. Tigecycline’s activity was evaluated by a study on *Escherichia coli* derivatives containing plasmids expressing different specific efflux genes (tet[B], tet[C], and tet[K]). An unchanged MIC value confirmed tigecycline’s protection against these efflux genes [90]. Moreover, glycylcyclines also manifest resistance to less common mechanisms, such as altered target site conformation, enzymatic degradation, and mutations in DNA gyrase [87].

Similar to tigecycline, omadacycline possesses excellent activity against bacterial isolates carrying a wide variety of resistance mechanisms, including both tet[K] and tet[O] genes simultaneously [22,91]. Due to the reversible binding of tetracyclines to ribosomes, they act as bacteriostatic agents. Instead, in vitro omadacycline has demonstrated bactericidal activity against *Haemophilus influenzae*, *Streptococcus penumoniae*, and *Moraxella catarrhalis* [78]. Omadacycline has no significant effect on the synthesis of RNA, DNA, and peptidoglycan. Like tigecycline, omadacycline binds to the 30S subunit of the bacterial ribosome with enhanced binding based on other molecular interactions [77]. Having a D-ring modified with a pyrrolidinacetamide side chain, eravacycline was designed to maintain its activity against resistant bacteria (e.g., carbapenem-resistant, MDR, and extended-spectrum cephalosporin-resistant Enterobacteriaceae and extended-spectrum, β-lactamase-producing Enterobacteriaceae) [41,92]. Eravacycline has 10 times the affinity for the ribosomal target in vitro and inhibits translation at four times lower concentrations than tetracycline [93]. A study conducted by *Batool* et al. in 2020 concluded that sarecycline differs slightly from other tetracycline derivatives in terms of mechanism of action, emphasising its unique role in this large family, a role that clinicians should take into account when evaluating its therapeutic potential. By analysing the crystal structure of sarecycline related to the bacterial initiation complex, it has demonstrated that in addition to binding to the same site of the small ribosomal subunit, sarecycline, due to the C7 moiety, expands and establishes uncommon interactions with mRNA. This contact leads to the stabilisation of the substance on the ribosome and an increased inhibitory effect. Thus, sarecycline overcome the mechanisms of bacterial resistance to tetracyclines [42,57].

#### Other Biological Effects

In addition to the approved therapeutical uses as antibiotics, tetracyclines have other biological effects, which have been investigated and exploited. These non-antibiotic properties comprise anti-inflammatory effects; anti-apoptotic activity; immunomodulatory properties; inhibitory effects on proteolysis, angiogenesis, and tumour metastasis; and a neuroprotector effect [17].

The anti-inflammatory activity of this class is mediated by a large number of mechanisms such as inhibition of neutrophil activation and migration; T lymphocyte activation and proliferation; inhibition of phospholipase, angiogenesis, nitric oxide synthesis, and granuloma formation; suppression of inflammatory cytokine release (TNFα, IL-1β, IL-6, IL-8); and decrease of reactive oxygen species [94,95]. However, the best-known mechanism of anti-inflammatory action is the inhibition of matrix metalloproteinases (MMPs). This mechanism occurs both directly and indirectly by inhibiting the MMPs synthesis [96]. Therefore, tetracyclines, such as doxycycline, minocycline, and sarecycline, are frequently prescribed for acne vulgaris when topical treatment is unsuccessful [94]. In a study using the carrageenan-induced rat paw oedema inflammation model, sarecycline presented an anti-inflammatory effect comparable to doxycycline and minocycline at all the tested doses [42]. Moreover, sarecycline has been proven to be a valuable alternative for treating papulopustular rosacea in terms of efficiency, safety, and tolerability [97].

The non-antibiotic properties of tetracyclines and their analogues were studied in both dermatological and non-dermatological diseases, as presented in Table 3 [98].

Research has shown that minocycline has several non-antibiotic biological effects that are beneficial in experimental models of various inflammatory diseases. These include dermatitis, periodontitis, atherosclerosis, and autoimmune diseases such as rheumatoid arthritis and inflammatory bowel disease. Due to its high lipophilicity, minocycline readily penetrates the blood–brain barrier and achieves high concentrations in the brain; hence, it has been effective in neuroprotection. However, because of its lipophilicity, vestibular side effects such as dizziness and vertigo have been associated with minocycline therapy. This outcome has been confirmed by experimental studies of ischemia, traumatic brain injury, and neuropathic pain, as well as several neurodegenerative diseases (e.g., Parkinson’s disease, Huntington’s disease, Alzheimer’s disease, amyotrophic lateral sclerosis, spinal cord injury, and multiple sclerosis). In addition, other pre-clinical studies have shown the ability of minocycline to inhibit malignant cell growth and activation, HIV replication, and prevent bone resorption [17].

### 3.4. Spectrum of Antibacterial Activity

Detailed antibacterial spectrum of the new generation of tetracyclines is shown in Table S5 (Supplementary Materials).

Tigecycline presents activity against MDR pathogens, such as MRSA; *Staphylococcus epidermidis*; vancomycin-resistant Enterococcus; *Acinetobacter* spp.; *Stenotrophomonas maltophilia*; penicillin-resistant *Streptococcus pneumoniae*; and Enterobacteriaceae resistant to aminoglycosides, carbapenems, fluoroquinolones, and β-lactamase producers [118,119]. Tigecycline is very active against *Neisseria gonorhoae* and *Eikenella corrodens*, as well as on rapidly growing species of mycobacteria (*M. chelonae*, *M. abscessus*, *M. fortuitum)* [120]. Although against most of tigecycline’s action is bacteriostatic, there are pathogens on which it acts bactericidally, such as *Legionella pneumophila* and *Streptococcus pneumoniae*. However, tigecycline is not effective against *Pseudomonas aeruginosa*, *Proteus mirabilis*, *Providencia* spp., or *Morganella morganii* [121].

Omadacycline is very potent against atypical bacteria, as well as Gram-positive and Gram-negative aerobic pathogens [22,122,123], and further against anaerobic bacteria that cause infections from dog or cat bites (except *Eikenella corrodens*); however, omadacycline is not effective on the species of *Proteus, Providencia*, *Morganella*, and *Pseudomonas* [124]. Generally, omadacycline acts as bacteriostatic agent, but against *Escherichia coli, Streptococcus pneumoniae*, and *Haemophilus influenzae* acts bactericidal [125]. This new tetracycline is more active than tigecycline and acts similar to eravacycline against Gram-positive pathogens [40].

Eravacycline is a broad-spectrum tetracycline that has showed a great activity against aerobic and anaerobic Gram-negative and Gram-positive bacteria, except *P. aeruginosa* and *Burkholderia cenocepacia*. Eravacycline also shows good activity against MDR bacteria, including Enterobacteriaceae and *A. baumannii*, expressing extended spectrum β-lactamases, carbapenem resistance, and mechanisms conferring resistance to other antibiotic classes [18]. Eravacycline is more effective than omadacycline against Gram-negative and broad-spectrum beta-lactamase-producing bacteria. Eravacycline is two to four times more active than tigecycline on clinically relevant Gram-positive species [40].

The spectrum of sarecycline is narrow, with little activity against aerobic and anaerobic Gram-negative bacteria and microflora commonly found in the gastrointestinal tract. The activity of this compound specifically targets *Cutinebacterium acnes*, but also some clinically relevant Gram-positive bacteria, including MRSA [126]. It is noteworthy that the prolonged and intermittent use of broad-spectrum antibiotics such as doxycycline and minocycline in acne vulgaris has been associated with the development of antimicrobial resistance and permanent perturbation of the gut and cutaneous microbiome. Although no causal relationship has been definitively established, the use of doxycycline in patients with acne was found to be associated with a 2.25-fold greater risk of developing Crohn’s disease [127,128].

### 3.5. Bacterial Resistance to New Tetracyclines

The widespread use of these antibacterial agents has unavoidably led to the development of bacterial resistance through plasmid-encoded tetracycline resistance genes (tet), conjugated transposons and integrons, which allow tet genes to be transmitted from one species/generation to another species/generation through conjugation [13,72,129]. The four main types of tetracycline resistance are outlined in Table 4 [18,27,38].

The expression of these genes leads to the production of proteins that contribute to the two primary mechanisms of resistance: ribosomal protection by dissociating tetracyclines from their target (e.g., tet[M], tet[O]) and the efflux of the substance out of the cell by active transport (e.g., tet[A], tet[B]) [28,118]. The efflux pumps are located in the cytoplasmic membrane and act through the antiport of a proton in exchange with a tetracycline–magnesium monocationic complex. Thus, the intracellular concentration of tetracyclines decreases [13]. The most common pumps are part of the Major Facilitator Superfamily of carriers [75]. In contrast to efflux pumps, for which the mechanism is elucidated, the ribosomal protection mechanism is not fully known. However, studies suggest that genes involved (e.g., tet[M], tet[O], and tet[S]) alter the conformation of ribosomes and displace the drug from the active site [38,85,130]. These genes are protein molecules GTPases (guanosine triphosphatases) that have structures and sequences similar to elongation factors (EF-G and EF-Tu) [131].

The other two less common mechanisms include two distinct genes that modify tetracyclines, leading to their degradation and mutations of the ribosomal 16S subunit, decreasing the affinity of the compounds for the ribosomal target [27]. The first of the mechanisms, chemical inactivation, is caused by a FAD-monooxygenase encoded by the tet[X] and tet [37] genes. These hydroxylate the C11 position, altering its structure and coordination with the magnesium ion, and therefore the affinity for the ribosome [38,132]. Moreover, the hydroxylated version degrades even in the absence of enzymes [133]. Because monooxygenase uses NADPH and O_2_ in its activity, this resistance occurs only in aerobic organisms [27]. The second mechanism of resistance, the site-binding mutation, is found predominantly in bacteria with a small number of copies of rRNA [18]. The first case was described by Ross et al. (1998) on a bacterial strain of *Propionibacterium acnes*. In addition, there are innate resistance mechanisms, with some bacteria being more immune to the tetracycline class due to differences in membrane permeability. For example, Gram-negative bacteria are more resistant due to the outer wall containing lipopolysaccharide molecules [27].

Although tigecycline is not affected by the main resistance mechanisms, since its introduction in the clinic (2005), the number of pathogens that have developed resistance is continuously increasing. In Gram-negative bacteria, most cases were caused by overexpression of resistance-nodulation-cell division (RND) pumps. For example, MexXY-OprM for *Pseudomonas aeruginosa* strains and AdeIJK/AdeABC for *Acinetobacter baumannii* [134,135,136,137]. Something similar occurs for some Gram-positive bacteria by overexpressing the Multidrug and Toxic Compound Extrusion (MATE) (case of MepA pump for Staphylococcus aureus) [138]. Other mechanisms also decrease the susceptibility of tigecycline to other microorganisms: mutations in ribosomal protein genes, mutations in the 16S subunit of rRNA [139], inactivation by FAD-dependent monooxygenase [140], and tetX gene-carrying plasmids [141]. A recent study on *Acinetobacter baumannii* found a resistance plasmid containing the tet(X5) gene, with similarity regarding structure and function of other tet(X) variants, probably using the same transfer elements for spreading [142].

The antimicrobial activity of omadacycline is not disturbed by the two significant resistance mechanisms in therapy [143]. This aspect is due to the functional groups in the C7 (dimethylamino) and C9 (aminomethyl) positions, which prevent the expulsion of the antibiotic by the efflux pumps and the ribosomal protection, respectively [22]. Omadacycline has demonstrated in vitro activity against bacterial strains containing efflux and ribosomal protection genes (*Staphylococcus aureus* expressing the tet(K) and tet(M) genes, *Enterococcus faecalis* expressing the tet(L) and tet(M) genes) [125]. However, the activity of omadacycline is decreased by mutations in the ribosomal RNA of some microorganisms. According to a study conducted by Heidrich et al. (2016), the MIC for tetracycline, tigecycline, and omadacycline on species containing mutations (G1055C, G996U) of the 16S ribosomal subunit increased four to eightfold, suggesting that they are susceptible to these changes, regardless to their affinity for the target [27,144]. The omadacycline’s action and the action of glycylcyclines, in general, are also affected by chemical inactivation [27].

Eravacycline avoids tet(A) efflux pumps, maintains activity against staphylococci-containing tet(K) genes, and successfully binds to bacterial ribosomes modified by tet(M) proteins [98,145]. However, eravacycline remains vulnerable to overexpression of MDR efflux pumps belonging to Gram-negative bacteria, change in ribosomal target (16S or 10S), and enzymatic degradation sometimes encountered in Bacterioides spp. The resistance to eravacycline has also been observed in mutant species of Enterococcus, mutations encoded by the rpsJ gene [41,146,147].

In the case of sarecycline, the probability of inducing bacterial resistance is low due to the narrow spectrum of activity and the unique structural modifications at the C7 position. The rate of spontaneous mutations varies from 10^−^^9^ to 10^−^^11^ in *Cutinebacterium acnes*, and 10^−^^9^ and 10^−^^8^ in *Staphylococcus aureus* and *Staphylococcus epidermidis*, respectively (at increases in MIC values between four and eight times) [126,148].

## 4. Therapeutic Use of the New Tetracyclines

In the past, tetracyclines have been widely used for various genitourinary, gastrointestinal, respiratory tract, and dermatological diseases. However, the tremendous onset of bacterial resistance, as well as the emergence of new antibacterial agents, has diminished the area of infections for which tetracyclines are considered the first therapeutic option [1,18,27].

The activity of tigecycline has been evaluated in several in vivo clinical trials in human subjects, following which the three FDA-approved indications were formulated. In the treatment of hospitalised patients with complicated skin and soft tissue infections, tigecycline has not only been shown to be effective but has also shown a favourable pharmacokinetic profile [149]. In another phase 2 open-label clinical trial, which included patients with complicated intra-abdominal infections (gangrenous perforated appendicitis, cholecystitis, diverticulitis, peritonitis), tigecycline was a safe and effective treatment [120]. Last but not least, tigecycline is indicated and approved in the treatment of community-acquired pneumonia. For all research, tigecycline met all non-inferiority criteria [31,150].

Omadacycline, unlike tigecycline or eravacycline, brings an advantage through oral formulation, facilitating patient compliance and hospitalisation costs [38]. Omadacycline is recommended in treating complicated skin and soft tissue infections and community-acquired pneumonia, but there are ongoing studies for its use in urinary tract infections [39,151,152,153].

Eravacycline, due to its broad antibacterial spectrum, in vitro activity, and superior tolerability in comparison with tigecycline, is an appropriate solution for treating complicated intra-abdominal infections in adults, especially when the pathogen possesses resistance mechanisms to other tetracyclines or classes of antibiotics [41]. Infection, for which the efficacy of eravacycline has been studied by comparison with the beta-lactam antibiotics meropenem or ertapenem, include: appendicitis, cholecystitis, diverticulitis, gastric or duodenal perforation, intra-abdominal abscess, intestinal perforation, and peritonitis [145].

The FDA-approved sarecycline is used for treating acne vulgaris in patients aged nine years or above, demonstrating efficacy against moderate-to-severe, inflammatory, or non-inflammatory (comedones) forms. In addition, due to its targeted action on *Cutinebacterium acnes* and low blood–brain penetration, sarecycline has a good safety profile (minimal side effects), low potential to induce bacterial resistance, and also potentially low impact on the gut microbiota when compared to the broad-spectrum doxycycline and minocycline [154,155].

*Pharmacokinetic properties.* Tigecycline is highly bound to plasma proteins and has a large volume of distribution (above plasmatic volume), which indicates its concentration in tissues. Moreover, tigecycline is rapidly distributed in tissues; the highest concentrations were observed in the bone marrow, thyroid gland, salivary glands, spleen, and kidneys. Tigecycline metabolises independent of cytochrome P450 enzymes, but not extensively. Consequently, tigecycline does not interfere with the metabolism of other substances mediated by the six cytochrome P450 isoforms (1A2, 2C8, 2C9, 2C19, 2D6, and 3A4) [31]. The pharmacokinetics of tigecycline is linear—this may be influenced by the coadministration of P-glycoprotein inhibitors or inducers; tigecycline acts as a substrate of these [156]. Similarly, omadacycline presented a low probability of interactions through transport mechanisms [133]. The omadacycline rate of absorption decreases if a high-fat meal is consumed two hours earlier. Thus, omadacycline must be taken after a fasting period of at least 4 h, followed by 2 h without ingestion of drinks and food (apart from water), as well as 4 h without administration of antacids, multivitamins, and dairy products [157]. The liver metabolises eravacycline, but none of the metabolites are pharmacologically active. Therefore, caution is required in CYP3A4 inducers to increase the extent of eravacycline metabolism to a clinically relevant rate [41]. Sarecycline inhibits P-glycoprotein in vitro. Consequently, decreasing dose and toxicity examination is required when it is coadministered with substrate substances [56]. Generally, the pharmacokinetics of modern tetracyclines are not remarkably influenced by age, sex, or renal function (including renal failure and haemodialysis) [29,39,158]. The pharmacokinetic parameters of the four modern tetracyclines are shown in Table S6 (Supplementary Materials).

## 5. Side Effects of the Third-Generation Tetracyclines

As an antibiotic class, tetracyclines are generally well tolerated. However, there is a diversity of side effects and contraindications, with these compounds affecting several systems of the human body. For example, tetracyclines often cause gastrointestinal disorders such as abdominal discomfort, nausea, vomiting, and epigastric pain. Moreover, typical side effects are photosensitivity, manifested by erythema and skin blisters, discolouration of the teeth, and inhibition of bone growth in children. Rarely, tetracyclines may cause increased intracranial pressure (pseudotumor cerebri), renal toxicity, hepatotoxicity, and *Clostridium difficile* infections [159].

Similar to tetracyclines in the first generations, the most common side effects of modern tetracyclines are those of the gastrointestinal tract [75,160,161,162]. Other side effects of the tetracycline new generation are shown in Table 5. Nausea and vomiting may occur in the first two days of treatment and are usually mild to moderate. In the case of tigecycline, these effects are correlated with the dose administered, the highest tolerated doses being 100 mg for healthy subjects without fasting, 200 mg postprandial [156]. Diarrhoea has been reported, and is associated, in the vast majority of cases, with *Clostridium difficile* superinfection, ranging from mild forms to severe or fatal colitis. Other less common side effects are constipation, anorexia, dyspepsia, dry mouth, acute pancreatitis, and pancreatic necrosis [1,31,39,46,145]. Regarding the pharmacotoxicology of sarecycline, due to its narrower spectrum, it does not affect the intestinal flora as much, and therefore adverse effects such as diarrhoea and fungal infections have been observed less clinically [126].

Tetracyclines affect teeth and bones by forming stable complexes with calcium ions, accumulating in deposits at these levels. Thus, the teeth may acquire a yellow to brown colour, sometimes even permanent, due to the formation of chelates of tetracycline-calcium orthophosphate, which darkens after exposure to the sun [126]. This phenomenon has effects from an aesthetic point of view but can be aggravated, leading to demineralisation and hypoplasia of tooth enamel with decreased resistance to caries attack [165]. Tooth staining is more common in long-term treatment with tetracycline derivatives but has also been observed with repeated short-term administrations. Children who receive tetracyclines in the first part of life and children whose mothers have used them since the second trimester of pregnancy tend to have tetracyclines deposited at the level of baby teeth. There was also a decrease in the rate of fibula growth and ossification processes for the foetus exposed in utero due to accumulation in the tissues [39,46,145].

Another subgroup of side effects is skin damage. Tetracyclines may cause allergic-type side effects with pruritus, transient rash, or itchy skin and hyperhidrosis [31,145]. These reactions are due to the increased sensitivity of the skin to light during systemic tetracycline therapy. Therefore, patients undergoing treatment should avoid excessive exposure to natural or artificial sunlight (ultraviolet radiation) [46]. Hypersensitivity reactions (Stevens–Johnson syndrome, anaphylaxis), sepsis, and death have been reported with low frequency when using tigecycline [166].

Hepatobiliary disorders due to tetracyclines are not very common in this class but are reflected in increased plasma concentrations of aspartate aminotransferase (AST), alanine aminotransferase (ALT), bilirubin, and hepatic transaminases (TGP, TGO). Other laboratory parameters that may change are increased amylase, lipase, gamma-glutamyltransferase, urea nitrogen (class effect), creatinine phosphokinase, alkaline phosphatase, and decreased creatinine clearance. These reactions occur relatively infrequently, with a frequency <2% for tigecycline and omadacycline, and <1% for eravacycline (following clinical trials) [31,39,145]. Cases of cholestatic jaundice and mild pancreatitis induced by tigecycline have been reported [73,167]. At the vascular level, forms intended for the intravenous route produce reactions at the site of administration. With the exception of sarecycline (orally administration), cases of extravasation of the infusion solution, hypoesthesia, pain, erythema, swelling, inflammation, irritation, phlebitis, and thrombophlebitis have been reported [29,39,145,168]. Other side effects that may occur with omadacycline are cardiovascular, with grouped clinical trials showing a frequency of over 2% of hypertension and <2% of tachycardia and atrial fibrillation. No adverse cardiovascular reactions are known for tigecycline. A study in healthy subjects showed that this compound has no significant effect on the QT interval [169].

At the haematological and lymphatic levels, tigecycline and omadacycline may lead to anaemia but have opposite effects on platelets, with tigecycline decreasing their number (thrombocytopenia) [170], compared to omadacycline, which may cause thrombocytosis [157]. Other common side effects of tigecycline are prolongation of partially activated thromboplastin time (aPTT) and prothrombin time. However, an increase in the international normalised ratio (INR) is less common [31].

The primary concern with tigecycline is the increased mortality due to its use, compared with other anti-infective agents [73]. The results obtained in phases 3 and 4 of 13 clinical trials alerted the FDA, which issued a black box warning about the increased risk of mortality of patients treated with this drug [171]. Consequently, several meta-analyses of all controlled and randomised clinical trials were performed, concluding that tigecycline is not indicated in severe infections and should only be reserved for use in situations where alternative treatments are not appropriate [170,172,173,174]. Because these studies also found higher rates of clinical failure, superinfections, and septic shock compared to the comparator group, several hypotheses were postulated in an attempt to find a cause. These could be low efficacy, low plasma concentrations (could explain persistent bacteremia), and low alveolar concentrations (partly explains the low efficacy in patients with pneumonia associated with mechanical ventilation) [175,176]. In addition, on the basis of animal studies (rats), eravacycline and omadacycline have been shown to have undesirable effects on fertility, affecting sperm production, maturation, morphology, and motility [39,145].

During pregnancy, tetracyclines are not recommended, due to fetotoxicity and teratogenicity (tigecycline, according to the recommendations given by the FDA is classified as risk category D). Tetracyclines are used only when the benefit to the mother outweighs the potential risk for the foetus [171]. The results of animal studies indicate that tetracyclines cross the placenta, reach therapeutic concentrations in the foetal circulation, and may have toxic effects on foetal growth (often related to delayed skeletal development) [43,177]. Cases of embryotoxicity in animal models treated at the beginning of pregnancy were also highlighted. In addition, tetracyclines are excreted in human milk. Although the rate of absorption for infants is unknown, it is recommended that they not be used in breastfeeding either to avoid the risk of tooth discolouration and damage to osteogenesis [31,39,46,145].

The absorption of modern oral tetracyclines (omadacycline, sarecycline), similar to older members of this class, may be affected by the concomitant use of multivitamins; antacids (containing aluminium, calcium); or those in the composition of which magnesium, iron, and/or zinc are found. In these situations, non-absorbable chelating complexes are formed. Tetracyclines may also increase the anticoagulant effect of warfarin [13]. Several reports note decreased coagulation efficiency and bleeding in some patients after starting tetracycline therapy [38]. In a pharmacokinetic-pharmacodynamic study, tigecycline decreased the clearance of warfarin, and therefore careful monitoring of anticoagulant levels is indicated by more frequent international normalised ratio (INR) and prothrombin time (PT) [31,52,178]. The use of tetracyclines may decrease the effectiveness of oral contraceptives, but this interaction is quite controversial due to limited information [38,179]. There is no clinically significant effect of sarecycline on the efficacy of oral contraceptives containing ethinyl estradiol and norethindrone acetate [46]. Other interactions recorded in the literature are increased serum digoxin concentration, interference with penicillin activity, and a synergistic effect with oral retinoids on increased intracranial pressure [38,46].

## 6. New Compounds under Development

**Figure 13:**
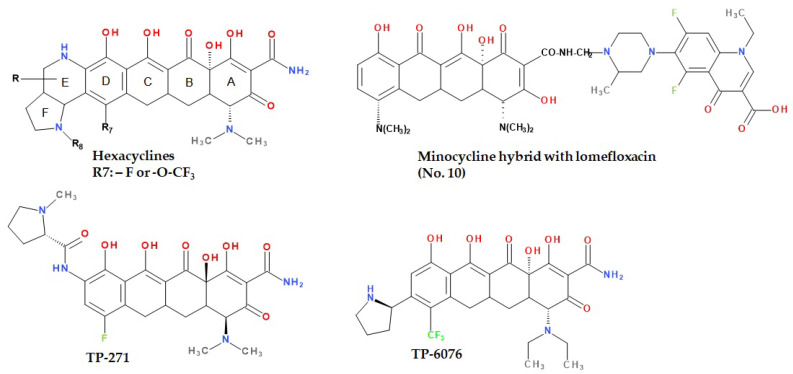
Promising derivatives of tetracyclines.

Sriram et al. (2007) synthesised tetracycline derivatives with anti-HIV, antimycobacterial, and HIV-1 integrase inhibitory properties. This was achieved by the reaction between certain tetracyclines (minocycline, tetracycline, and oxytetracycline), formaldehyde, and the secondary amine function (piperazine) of some fluoroquinolones (norfloxacin, lomefloxacin, ciprofloxacin, gatifloxacin), with the help of microwave radiation. Compound no. 10 (a tetracycline hybrid with lomefloxacin) represented in Figure 13 demonstrated the most potent effect on HIV-1 replication. These studies show that the combination of tetracyclines with fluoroquinolones has resulted in both anti-HIV and anti-tuberculosis activity (*Mycobacterium tuberculosis*) and has a promising prospect in treating AIDS [180,181].

Using total synthesis, Sun et al. (2015) projected a series of tetracycline analogues with six fused rings called hexacyclines. Their structure consists of the classical skeleton of tetracyclines, having attached a bicyclic ring EF at the level of ring D (Figure 13) [170].

The relationships between chemical structure and antibacterial activity were tested, with substitutions in positions C7, N8, C9, and C10, evaluating the efficacy of various analogues on a wide range of Gram-positive and Gram-negative bacteria, including tetracycline-resistant or multidrug-resistant strains. Of all the compounds studied, the best results were recorded for C7-fluorohexacycline and C7-trifluoromethoxyhexacycline, with a broad antibacterial activity in vitro and good activity in vivo on *Pseudomonas aeruginosa*. The promising data extracted from this study support the optimisation of this type of skeleton to discover and obtain in the future some new tetracyclines that are clinically valuable [182].

Currently, Tetraphase Pharmaceuticals holds two compounds, phase I of clinical trials, TP-271 and TP-6076, whose structures are represented in Figure 13 [183,184,185,186]. Thanks to a research program opened in the mid-1990s, synthetic routes with increased scalability and efficacy of obtaining tetracycline analogues have been discovered. To date, more than 3000 analogues have been synthesised using these methods, including the two previously mentioned [187]. TP-271 is a new, clinically developing fluorocycline with promising activity against bacteria that cause respiratory infections, community-acquired pneumonia, anthrax, bubonic plague, and tularemia [187]. Following both in vivo studies and in vivo evaluations, TP-271 has shown an increased potential against susceptible and multidrug-resistant pathogens associated with moderate to severe community-acquired pneumonia. These include key bacteria in respiratory infections, *Streptococcus pneumoniae* (MIC_90_ = 0.03 μg/mL), methicillin-sensitive *Staphylococcus aureus* (MIC_90_ = 0.25 μg/mL), methicillin-resistant *Staphylococcus aureus* (MIC_90_ = 0.12 μg/mL), *Streptococcus pyogenes* (MIC_90_ = 0.03 μg/mL), *Moraxella catarrhalis* (MIC_90_ ≤ 0.016 μg/mL), and *Haemophilus influenzae* (MIC_90_ = 0.12 μg/mL) [185]. TP-271 has also shown strong activity against important pathogens: *Yersinia pestis*, *Bacillus anthracis*, *Francisella tularensis, Burkholderia mallei*, and *Burkholderia pseudomallai* [188]. Furthermore, TP-271 has been shown to be effective in animal studies of immunocompetent pneumonia and neutropenia with *Streptococcus pneumoniae*, MRSA, and *Haemophilus influenzae*. Regarding the mechanism of action, this compound binds to the 30S ribosomal subunit and maintains its activity, even in the presence of the ribosomal protective protein Tet (M). Therefore, due to the positive results obtained on animal models and the broad spectrum, TP-271 is a promising candidate for treating moderate to severe community-acquired pneumonia [185].

Tetraphase Pharmaceuticals investigated analogues of C4, C7, and C8 trisubstituted tetracyclines. Thereby, some of these tetracyclines have demonstrated increased in vitro potency against clinically significant pathogens, including *Acinetobacter baumannii* (MIC_90_ = 0.063 μg/mL) and carbapenem-resistant Enterobacteriaceae (MIC_90_ = 0.5 μg/mL). The C4-positioned diethyl-amine analogue, TP-6076 (Figure 13), currently in phase I of clinical trials, showed the highest activity of all the three-substituted compounds analysed. The phase I clinical trial is focused on pharmacokinetics and safety studies to assess the bronchopulmonary disposition of intravenous TP-6076 in healthy subjects, in order to assess the potential utility in *Acinetobacter baumannii* pneumonia. According to Tetraphase, TP-6076 is in development for the treatment of serious and life-threatening bacterial infections. The potency of this compound on Gram-negative, multidrug-resistant bacteria is 2 to 64 times higher than that of tigecycline. TP-6076 also retains a high efficacy against isolates with intrinsic resistance mechanisms that generally affect the tetracycline class (e.g., carbapanemase-producing Enterobacteriaceae and carbapanemase-producing *Acinetobacter baumannii*). Antibacterial activity was not affected by the type of carbapenem resistance determinant or international clone. The increased in vitro potency also resulted in high in vivo efficiency in models of mice infected with resistant Gram-negative multi-drug isolates [185,189,190].

## 7. Conclusions

The evolution of the tetracycline class is remarkable, through its development of semisynthetic analogues of the second generation, and, more recently, of the third generation. The new tetracyclines had acquired high potency and increased efficacy, even against resistant bacteria to tetracyclines. On the basis of the classical method of production, biosynthesis, and semi-synthesis, we are able to obtain the new compounds by total chemical synthesis, such as eravacycline.

The main focus in optimising the chemical structure was on modifying the C7 and C9 positions of the D ring in the simplest tetracycline with biological activity (sancycline). Thus, a new tetracycline class was discovered, based on C9-aminotetracyclines, which bear a glycyl moiety known as glycylcyclines. First in class was the tigecycline representative. Recently approved tetracyclines include beside tigecycline, omadacycline (an aminomethylcycline), eravacycline (a fluorocycline), and sarecycline (a 7-[(methoxy-(methyl)-amino)-methyl]methyl] derivative).

Tigecycline has the advantage of a superior potency over Gram-positive and Gram-negative MDR bacteria; its pharmaceutical formulation is only parenteral. Omadacycline has a broad spectrum of activity, including MRSA, penicillin-resistant, MDR *Streptococcus pneumoniae* and vancomycin-resistant enterococci. This new tetracycline drug is more advantageous in therapy than tigecycline because it can be administered orally and parenterally. Eravacycline is a synthetic fluorocycline with a great activity against Gram-positive and Gram-negative bacteria that developed specific resistance mechanisms to tetracyclines. Similar to tigecycline, eravacycline is administered exclusively parenterally. The main advantage of sarecycline is the narrow-spectrum activity and the higher selective activity against *Cutinebacterium acnes*. Sarecycline is available as an oral formulation to treat inflammatory lesions of moderate-to-severe non-nodular acne vulgaris.

Although tetracyclines currently act bacteriostatically, omadacycline has demonstrated bactericidal activity in vitro against some bacterial agents. It was proven that glycylcyclines manifest resistance to less common mechanisms, such as altered target site conformation, enzymatic degradation, and mutations in DNA gyrase. Therefore, eravacycline was designed to maintain its activity against resistant bacteria. Sarecycline expands and establishes uncommon interactions with mRNA.

Currently, some studies confirm other biological effects in tetracyclines class that require in-depth future studies. Therefore, through its newly acquired members, this class of antibiotics arouses the interest of researchers in the field. Consequently, new derivatives have been already developed, and many are in development. These are studied primarily studied for the antibiotic effect and also for other biological effects.

## Figures and Tables

**Table 1:** Tetracyclines—classification into generations [14,21,22,23,24].

Generations	Obtaining Method	Representatives
First	Biosynthesis	Chlortetracycline, oxytetracycline, tetracycline, demeclocycline
Second	Semisynthesis	Doxycycline, minocycline, lymecycline, meclocycline, methacycline, rolitetracycline
Third	Semisynthesis	Tigecycline, omadacycline, sarecycline
	Total synthesis	Eravacycline

**Table 2:** Sarecycline microspecies and the degree of ionisation as a function of pH (calculated) [66].

No.	Microspecies	No.	Microspecies	No.	Microspecies
**1**	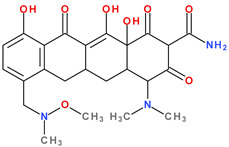	**2**	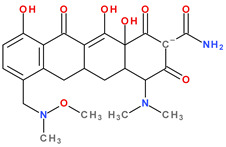	**4**	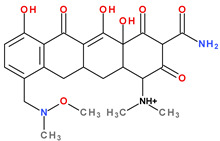
	pH 6.60 (32.92%; highest) pH 7.40 (17.87%)		pH 7.60 (22.31%; highest) pH 7.40 (21.59%)		pH 7.60 (12.59%; highest) pH 7.40 (12.19%)
**3**	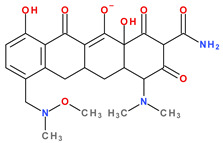	**5**	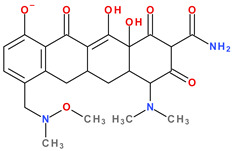	**6**	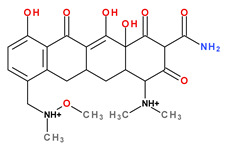
	pH 4.00 (98.86%; highest) pH 7.40 (2.11%)		pH 7.60 (3.84%; highest) pH 7.40 (3.72%)		pH 0.00 (98.16%; highest) pH 7.40 (0.00%)
**7**	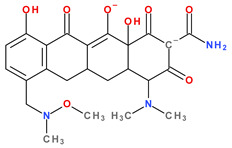	**8**	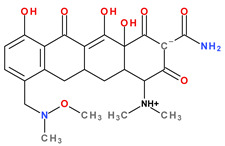	**9**	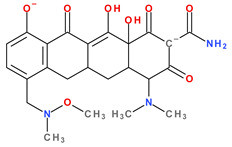
	pH 8.60 (49.38%; highest) pH 7.40 (11.90%)		pH 6.80 (24.07%; highest) pH 7.40 (13.10%)		pH 8.80 (17.75%; highest) pH 7.40 (4.50%)
**10**	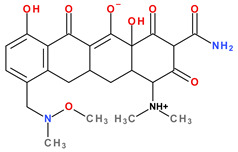	**11**	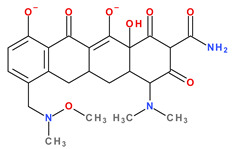	**12**	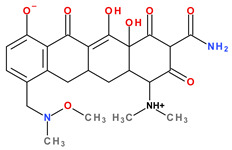
	pH 8.80 (17.75%; highest) pH 7.40 (4.50%)		pH 6.60 (2.80%; highest) pH 7.40 (1.52%)		pH 6.60-6.80 (0.81%; highest) pH 7.40 (0.44%)
**13**	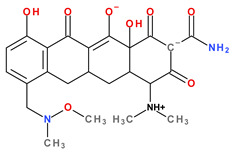	**14**	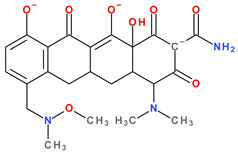	**15**	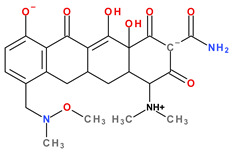
	pH 7.60 (7.85%; highest) pH 7.40 (7.60%)		pH 11.00 (96.30%; highest) pH 7.40 (0.26%)		pH 7.60 (2.82%; highest) pH 7.40 (2.73%)
**16**	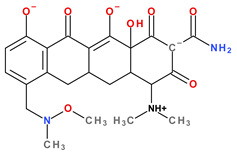	**17**	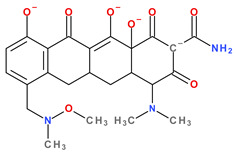		
	pH 8.60 (0.69%; highest) pH 7.40 (0.17%)		pH 14.00 (95.55%; highest) pH 7.40 (0.00%)		

**Table 3:** Other therapeutical uses of tetracyclines related to their non-antibiotic properties.

Dermatological Conditions	Reference	Non-Dermatological Conditions	Reference
Acne	[99,100]	Rheumatoid arthritis	[101,102]
Rosacea	[103,104]	Scleroderma	[105]
Bullous dermatitis	[106]	Cancer	[107]
Kaposi’s sarcoma	[108]	Aortic aneurysm	[109]
Sarcoidosis	[110]	Acute myocardial infarction	[111]
*Pyoderma gangrenosum*	[112]	Periodontitis	[113]
*Hidradenitis suppurativa*	[114]		
Sweet’s syndrome	[115]		
Alpha-1-antitrypsin deficiency panniculitis	[116]		
Pityriasis lichenoides chronica (PLC)	[117]		

**Table 4:** Mechanisms of resistance and resistance determinants of tetracyclines.

Resistance Determinants	Resistance Mechanisms			
	Efflux Pump	Ribosomal Protection	Chemical Inactivation	rRNA Mutations
Gram-positive bacteria	tetK, tetL, tetV, tetY, tetZ, tetAP, tet 33, tet 38, tet40, tet 45, otrB otrC, ter3	tetM, tetO, tetP, tetQ, tetS, tetT, tetW, tetZ, tetB(P), tet32, tet36, otrA	-	G1058C
Gram negative bacteria	tetA, tetB, tetC, tetD, tetE, tetG, tetH, tetJ, tetK, tetL, tetY, tet30, tet31, tet34, tet 35, tet39, tet41, tet42	tetM, tetO, tetQ, tetS, tetW, tet36, tet44	tetX, tet34, tet37	A926T, A928C, G927T, ΔG942, G966U

**Table 5:** Other side effects of modern tetracyclines [31,33,38,39,46,145,152,154,160,163,164].

No.	Affected Level/Disorders	Side Effects	Representatives (Frequency)
1	Nervous system	lethargy, dizziness, dysgeusia, tinitus, vertigo	Sarecycline (<1%)
2	Metabolism	hypocalcemia	Tigecycline (<2%) Eravacycline (<1%)
		hyponatremia, hypoglycemia	Tigecycline (<2%)
3	Psychiatric disorders	anxiety, insomnia, depression	Eravacycline (<1%) Tigecycline, omadacycline (insomnia only)
4	Urogenital disorders	vulvovaginal fungal infections, vulvovaginal candidiasis vaginal moniliasis, vaginitis, leukorrhea	Sarecycline, omadacycline (no data available)
		vaginal moniliasis, vaginitis, leukorrhea	Tigecycline (<2%)
5	Respiratory system	oropharyngeal pain	Omadacycline (<2%)
		pleurisy, dyspnea	Eravacycline (<1%)
6	Others	vertigo	Omadacycline (<2%)
		abdominal pain	Tigecycline (>2%), omadacycline (<2%)

## Supplementary Materials

The following are available online at https://www.mdpi.com/article/10.3390/pharmaceutics13122085/s1, Table S1. The representatives of tetracyclines class from first and second generations and their approval in therapy (FDA—USA Food and Drug Administration, EMA—European Medicine Agency, MHRA—UK Medicines and Healthcare Products Regulatory Agency). Table S2. Modern tetracyclines of the third generation introduced in therapy. Table S3. Essential structural features of tetracycline antibiotics. Table S4. Physicochemical properties of third-generation tetracyclines. Table S5. The antibacterial spectrum of the newly approved tetracyclines. Table S6. Pharmacokinetics parameters of modern tetracyclines.
