# Supplementary material for: The Development of Third-Generation Tetracycline Antibiotics and New Perspectives
Source: Pharmaceutics. 2021 Dec 5;13(12):2085. doi: 10.3390/pharmaceutics13122085 (PMC8707899; doi:10.3390/pharmaceutics13122085)
Supplement: Supplementary file 1 [file pharmaceutics-13-02085-s001.zip › pharmaceutics-1462294-supplementary.pdf]

pharmaceutics

MDPI

Supplementary Materials: The Development of Third-Generation Tetracycline Antibiotics and New Perspectives

Aura Rusu and Emanuela Lorena Buta

Table S1. The representatives of tetracyclines class from first and second generations and their approval in therapy (FDA – USA Food and Drug Administration, EMA – European Medicine Agency, MHRA - UK Medicines and Healthcare Products Regulatory Agency).

Generic name (Commercial name)	Generation	Year of approval	The authorizing entity	IUPAC name	Reference
**Chlortetracycline (Aureomycin)**	First	1948	FDA	(4*S*,4*aS*,5*aS*,6*S*,12*aR*)-7-chloro-4-(dimethylamino)-1,6,10,11,12*a*-pentahydroxy-6-methyl-3,12-dioxo-4,4*a*,5,5*a*-tetrahydrotetracene-2-carboxamide	[1–4]
**Oxytetracyline (Terramycin)**	First	1950	FDA	(4*S*,4*aR*,5*S*,5*aR*,6*S*,12*aR*)-4-(dimethylamino)-1,5,6,10,11,12*a*-hexahydroxy-6-methyl-3,12-dioxo-4,4*a*,5,5*a*-tetrahydrotetracene-2-carboxamide	[1,5]
**Tetracycline (Achromycin)**	First	1954	FDA	(4*S*,4*aS*,5*aS*,6*S*,12*aR*)-4-(dimethylamino)-1,6,10,11,12*a*-pentahydroxy-6-methyl-3,12-dioxo-4,4*a*,5,5*a*-tetrahydrotetracene-2-carboxamide	[1,6,7]
**Rolitetracycline (Reverin)**	First	1959	FDA	(4*S*,4*aS*,5*aS*,6*S*,12*aR*)-4-(dimethylamino)-1,6,10,11,12*a*-pentahydroxy-6-methyl-3,12-dioxo-*N*-(pyrrolidin-1-ylmethyl)-4,4*a*,5,5*a*-tetrahydrotetracene-2-carboxamide	[8,9]
**Demeclocycline (DMCT)**	First	1960	FDA	(4*S*,4*aS*,5*aS*,6*S*,12*aR*)-7-chloro-4-(dimethylamino)-1,6,10,11,12*a*-pentahydroxy-3,12-dioxo-4*a*,5,5*a*,6-tetrahydro-4*H*-tetracene-2-carboxamide	[10]
**Doxycycline (Vibramycin)**	Second	1967	FDA	(4*S*,4*aR*,5*S*,5*aR*,6*R*,12*aR*)-4-(dimethylamino)-1,5,10,11,12*a*-pentahydroxy-6-methyl-3,12-dioxo-4*a*,5,5*a*,6-tetrahydro-4*H*-tetracene-2-carboxamide	[1,11]
**Minocycline (Minocyn)**	Second	1971	FDA	(4*S*,4*aS*,5*aR*,12*aR*)-4,7-bis(dimethylamino)-1,10,11,12*a*-tetrahydroxy-3,12-dioxo-4*a*,5,5*a*,6-tetrahydro-4*H*-tetracene-2-carboxamide	[12]
**Methacycline (Rondomycin)**	Second	1982???	FDA	(4*S*,4*aR*,5*S*,5*aR*,12*aR*)-4-(dimethylamino)-1,5,10,11,12*a*-pentahydroxy-6-methylidene-3,12-dioxo-4,4*a*,5,5*a*-tetrahydrotetracene-2-carboxamide	[13,14]
**Lymecycline (Tetralysal)**	Second	1995	MHRA and EMA	(2*S*)-6-[[[(4*S*,4*aS*,5*aS*,6*S*,12*aR*)-4-(dimethylamino)-1,6,10,11,12*a*-pentahydroxy-6-methyl-3,12-dioxo-4,4*a*,5,5*a*-tetrahydrotetracene-2-carbonyl]amino]methylamino]-2-aminohexanoic acid	[15,16]

Table S2. Modern tetracyclines of the third generation introduced in therapy.

Compound name (Commercial name)	Year of approval	The authorizing entity	IUPAC name	Approved use in therapy	Reference
**Tigecycline (Tygacil)**	2005, 2006	FDA, EMA	(4*S*,4*aS*,5*aR*,12*aR*)-9-[[2-(tert-butylamino)acetyl]amino]-4,7-bis(dimethylamino)-1,10,11,12*a*-tetrahydroxy-3,12-dioxo-4*a*,5,5*a*,6-tetrahydro-4*H*-tetracen-2-carboxamide	Complicated skin and soft tissue infections<br>Complicated intra-abdominal infections<br>Community-acquired bacterial pneumonia	[17][18][19]
**Omadacycline (Nuzyra)**	2018	FDA	4*S*,4*aS*,5*aR*,12*aR*)-4,7-bis(dimethylamino)-9-[(2,2-dimethylpropylamino)methyl]-1,10,11,12*a*-tetrahydroxy-3,12-dioxo-4*a*,5,5*a*,6-tetrahydro-4*H*-tetracene-2-carboxamide	Complicated skin and soft tissue infections<br>Complicated intra-abdominal infections	[20]
**Eravacycline (Xerava)**	2018	FDA, EMA	(4S,4aS,5aR,12aR)-4-(dimethylamino)-7-fluoro-1,10,11,12a-tetrahydroxy-3,12-dioxo-9-[(2-pyrrolidin-1-ylacetyl)amino]-4a,5,5a,6-tetrahydro-4H-tetracene-2-carboxamide	Complicated intra-abdominal infections	[21,22]
**Sarecycline (Seysara)**	2018	FDA	(4S,4aS,5aR,12aR)-4-(dimethylamino)-1,10,11,12a-tetrahydroxy-7-[[methoxi(methyl)amino]methyl]-3,12-dioxo-4a,5,5a,6-tetrahydro-4H-tetracene-2-carboxamide	Moderate to severe non-nodular acne vulgaris	[23]

Table S3. Essential structural features of tetracycline antibiotics.

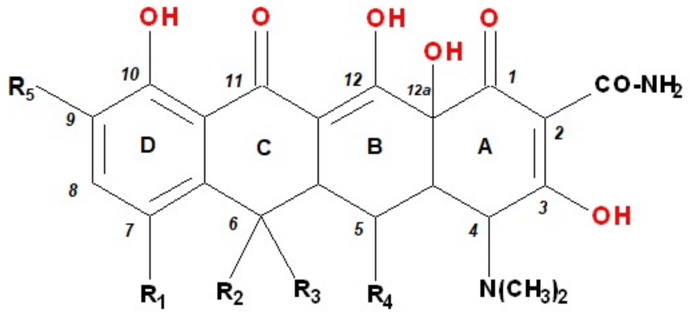

Tetracycline representatives	C9 (R_5)	C7 (R_1)	C6 (R_2)	C6 (R_3)	C5 (R_4)	C2
Chlortetracycline	-	**Cl**	OH	$-CH_3$	-	$-CO-NH_2$
Oxytetracycline	-	-	OH	$-CH_3$	OH	$-CO-NH_2$
Tetracycline	-	-	OH	$-CH_3$	-	$-CO-NH_2$
Rolitetracycline	-	-	OH	$-CH_3$	-	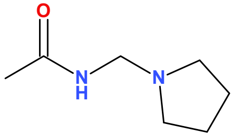
Demeclocycline	-	**Cl**	OH	-	-	$-CO-NH_2$
Metacycline	-	-	-	$=CH_2$	OH	$-CO-NH_2$
Doxycycline	-	-	-	CH_3	OH	$-CO-NH_2$
Minocycline	-	$-N(CH_3)_2$	-	-	-	$-CO-NH_2$
Limecycline	-	-	OH	$-CH_3$	-	$-CO-NH-CH_2-NH-(CH_2)_4-CH(NH_2)-COOH$
Sancycline	-	-	-	-	-	$-CO-NH_2$
Tigecycline	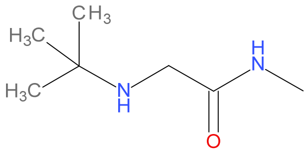	$-N(CH_3)_2$	-	-	-	$-CO-NH_2$
Omadacycline	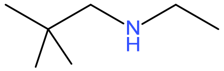	$-N(CH_3)_2$	-	-	-	$-CO-NH_2$

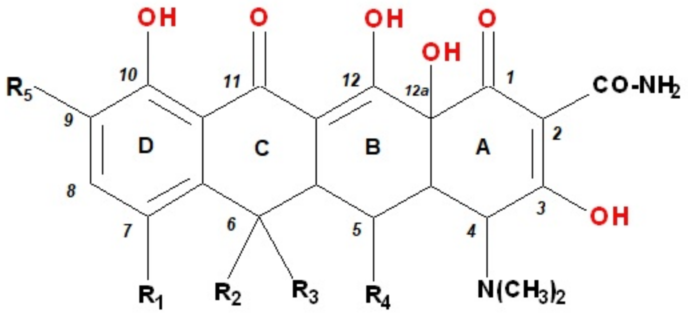

Tetracycline representatives	C9 (R_5)	C7 (R_1)	C6 (R_2)	C6 (R_3)	C5 (R_4)	C2
Eravacycline	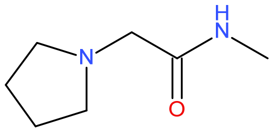	-F	-	-	-	-CO-NH_2
Sarecycline	-	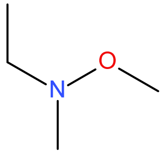	-	-	-	-CO-NH_2

Table S4. Physicochemical properties of third-generation tetracyclines.

Tigecycline	Properties	References
Chemical formula	$C_{29}H_{39}N_5O_8$	[24]
Molecular weight (g/mol)	586.6	[24]
Aspect	odourless, crystalline orange powder	[25]
Solubility	14.55 mg/L at 25°C (water); 0.45 mg/mL (water) (ALOGPS); highly ionisable throughout the pH range	[24,26]
LogP	0.8 (experimental), 0.66 (ALOGPS), -3.9 (ChemAxon)	[26]
p*K*a	3.17 (strongest acidic), 8.97 (strongest basic);<br>2.8; 4.4; 7.4; 8.9; 9.5	[26,27]
Melting point	163-173°C; decomposition at 188.6°C	[28,29]
Storage	2–8 °C; under –20 °C	[25,28]
**Omadacycline**	**Properties**	**References**
Chemical formula	$C_{29}H_{40}N_4O_7$	[30,31]
Molecular weight (g/mol)	556.66	[30,31]
Aspect	powder	[32]
Solubility	0.213 mg/mL (in water)	[30]
LogP	0.92 (ALOGPS), -2.2 (ChemAxon)	[30]
p*K*a	2.87 (strongest acidic), 10,54 (strongest basic) (Chemaxon)	[31]
Melting point	Not available	
Storage	under –20 °C	[32]
**Omadacycline (tosilate)**	**Properties**	**References**
Chemical formula	$C_{36}H_{48}$N4O10S	[33]
Molecular weight (g/mol)	728.85	[33]
Aspect	Not available	
Solubility	100 mg/mL at 25 ^{0}C (water, ethanol, [1] DMSO)	[33]
LogP	4.736	[34]
p*K*a	2.87 (strongest acidic), 10,54 (strongest basic) (Chemaxon)	[35]
Melting point	Not available	

Storage	three years at -20°C (powder)<br>two years at -80°C (in solvent)	[33]
**Eravacycline**	**Properties**	**References**
Chemical formula	$C_{27}H_{31}FN_4O_8$	[36]
Molecular weight (g/mol)	558,56	[36]
Aspect	Pale yellow to dark yellow cake	[37]
Solubility	0.838 mg/mL (water)(ALOGPS)	[38]
LogP	0.24 (ALOGPS), -3.5 (ChemAxon)	[38]
p*K*a	-2.96 (strongest acidic), 9 (strongest basic)	[38]
Melting point	Not available	
Storage	Not available	
**Eravacycline (dihydrochloride)**	**Properties**	**References**
Chemical formula	$C_{27}H_{33}Cl_2FN_4O_8$	[39]
Molecular weight (g/mol)	631.5	[39]
Aspect	Powder, pale yellow to dark yellow	[37]
Solubility	0.838 mg/mL (water)(ALOGPS); 50 mg/mL(water), 220 mg/mL ([1] DMSO);<br>Unstable in solutions, use freshly prepared.	[40,41]
LogP	0.24 (ALOGPS), -3.5 (ChemAxon)	[40]
p*K*a	-2.96 (strongest acidic), 9 (strongest basic)	[40]
Melting point	Not available	
Storage	-80°C; protected from light, stored under nitrogen	[41]
**Sarecycline**	**Properties**	**References**
Chemical formula	$C_{24}H_{29}N_3O_8$	[42]
Molecular weight (g/mol)	487.5	[42]
Aspect	Not available	
Solubility	2.01 mg/mL (water)(ALOGPS);	[43]
LogP	-0.17 (ALOGPS), -3.1 (ChemAxon)	[43]
p*K*a	3.31 (strongest acidic), 8.699 (strongest basic)	[43]
Melting point	Not available	
Storage	0-4°C (days to weeks; dry and dark place)<br>-20°C (months to years)	[44]
**Sarecycline (hydrochloride)**	**Properties**	**References**
Chemical formula	$C_{24}H_{30}ClN_3O_8$	[45]
Molecular weight (g/mol)	524.0	[45]
Aspect	Solid powder	[44]

Solubility	2.01 mg/mL (water)(ALOGPS); soluble in [1] DMSO	[44,46]
LogP	-0.17 (ALOGPS), -3.1 (ChemAxon)	[46]
p*K*a	3.31 (strongest acidic), 8.69 (strongest basic)	[46]
Melting point	Not available	
Storage	4°C (stored under nitrogen)	[47]

[1] DMSO—Dimethyl sulfoxide.

Table S5. The antibacterial spectrum of the newly approved tetracyclines.

Tetracycline	Gram-positive pathogens	Gram-negative pathogens	Other pathogens	References
**Tigecycline**	***Aerobes*** -**Cocci:** *Staphylococcus aureus,* coagulase-negative staphylococci, *Streptococcus pneumoniae, Enteroccocus faecalis, Enterococcus faecium, Enterococcus avium, Enterococcus casseliflavus, Enterococcus fallinarum, Enterococcus raffinosus,* Group A and B streptococci, Viridans streptococci ***Anaerobes*** **-Cocci:** *Peptostreptococcus* spp., **-Bacilli:** *Bacteroides fragilis, Clostridium perfringens, Clostridium difficile, Propionibacterium acnes, Fusobacterium* spp., *Prevotella* spp., *Porphyromonas* spp.	***Aerobes*** **-Bacilli:** -Enterobacteriaceae: *Escherichia coli, Klebsiella pneumoniae, Klebsiella oxytoca, Morganella morganii, Proteus mirabilis, Proteus vulgaris, Providencia* spp., *Shigella* spp., *Salmonella* spp., *Citrobacter freundi, Enterobacter cloacae, Enterobacter aerogenes, Serratia marcescens* -Non-Enterobacteriaceae: *Stenotrophomonas maltophilia, Pseudomonas aeruginosa, Burkholderia cepacia, Acinetobacter baumannii* (coccobacillus) -Respiratory pathogens: *Haemophilus influenzae* (coccobacillus), *Moraxella catarrhalis* (diplococcus) -Other: *Neisseria gonorrhoeae* (coccus), *Eikenella corrodens* (bacillus)	***Atypical bacteria:*** **-Bacilli:** *Mycobacterium abscessus, Mycobacterium chelonae, Mycobacterium fortuitum* group, *Mycobacterium avium* complex, *Mycobacterium lentiflavum, Mycobacterium marinum, Mycobacterium kansasii, Chlamydophyla pneumoniae,* -**Pleomorphic:** *Mycoplasma hominis, Mycoplasma pneumoniae, Ureaplasma urealyticum*	[48–71]
**Omadacycline**	***Aerobes*** **-Cocci:** *Staphylococcus aureus, Staphylococcus epidermidis,* coagulase-	***Aerobes*** **-Bacili:** *Escherichia coli, Klebsiella pneumoniae, Klebsiella oxytoca, Enterobacter*	***Atypical bacteria:*** **-Bacilli:** *Legionella pneumophila, Chlamydophila pneumoniae,*	[71–88]

Tetracycline	Gram-positive pathogens	Gram-negative pathogens	Other pathogens	References
	negative staphylococci, *Staphylococcus lugdunensis, Staphylococcus pseudintermedius, Enterococcus spp., Enterococcus faecium, Streptococcus pneumoniae, Streptococcus anginosus* group, Viridans streptococci, β-hemolytic streptococci, *Streptococcus pyogenes, Streptococcus agalactiae,*<br>**-Bacilli:** *Corynebacterium spp., Bacillus anthracis, Listeria monocytogenes*<br>***Anaerobes***<br>**-Cocci:** *Peptostreptococcus* spp.<br>**-Bacilli:** *Clostridium difficile, Clostridium perfringens,*	*cloacae, Citrobacter freundi, Proteus mirabilis, Salmonella* spp., *Serratia marcescens, Shigella* spp., *Yersinia pestis, Pseudomonas aeruginosa, Strenotrophomonas maltophilia, Burkholderia cepacia, Eikenella corrodens, Bergeyella zoohelcum*<br>**-Coccobacilli:** *Acinetobacter baumannii, Haemophilus influenzae, Pasteurella canis, Pasteurella multocida*<br>**-Diplococci:** *Moraxella catarrhalis, Neisseria gonorrhoeae, Neisseria weaveri, Neisseria zoodegmatis*<br>***Anaerobes***<br>**-Bacilli:** *Bacteroides fragilis, Bacteroides ovatus, Bacteroides thetaiotoamicron, Bacteroides vulgatus, Bacteroides pyogenes, Fusobacterium* spp., *Porphyromonas* spp., *Prevotella* spp.	*Mycobacterium abscessus, Mycobacterium fortuitum, Mycobacterium chelonae*<br>**-Pleomorphic:** *Mycoplasma hominis, Mycoplasma pneumoniae, Ureaplasma* spp.	
**Eravacycline**	***Aerobes***<br>**-Cocci:** *Staphylococcus aureus, Staphylococcus epidermidis, Streptococcus agalactiae, Streptococcus pneumoniae, Streptococcus pyogenes, Enterococcus faecalis, Enterococcus faecium*<br>***Anaerobes***<br>**-Cocci:** *Anaerococcus* spp., *Peptostreptococcus anaerobius, Peptostreptococcus micros*	***Aerobes***<br>**-Bacilli:** *Citrobacter freundii, Enterobacter aerogenes, Enterobacter cloacae, Escherichia coli, Klebsiella oxytoca, Klebsiella pneumoniae, Morganella morganii, Proteus mirabilis, Proteus vulgaris, Pseudomonas aeruginosa, Salmonella* spp., *Salmonella* spp., *Serratia marcescens, Strenotrophomonas maltophilia*<br>**-Diplococci:** *Moraxella catarrhalis, Neisseria gonorrhoeae*<br>**-Coccobacilli:** *Haemophilus influenzae*<br>***Anaerobes***<br>**-Bacilli:** *Bacteroides fragilis, Bacteroides ovatus, Bacteroides thetaiotoamicron, Fusobacterium* spp., *Prevotella bivia, Prevotella buccae,*		[71,89–93]

Tetracycline	Gram-positive pathogens	Gram-negative pathogens	Other pathogens	References
	**-Bacilli:** *Clostridium difficile, Clostridium perfringens, Lactobacillus* spp., *Propionibacterium* spp	*Prevotella disiens, Prevotella intermedia, Prevotella melaninogenica*		
**Sarecycline**	***Aerobes***	***Aerobes***		[94,95]
	**-Cocci:** *Staphylococcus aureus, Staphylococcus epidermidis, Staphylococcus haemolyticus, Streptococcus pyogenes, Streptococcus agalactiae, Enterococcus faecalis, Enterococcus faecium*	**-Bacilli:** *Enterobacter cloacae, Escherichia coli, Klebsiella pneumoniae, Proteus mirabilis, Salmonella* spp.		
	Anaerobes			
	**-Bacilli:** *Cutinebacterium acnes*			

Table S6. Pharmacokinetics parameters of modern tetracyclines (iv - intravenuos, AUC - aria under the curve, Cmax - maximum concentration observed, ss - steady state, D - dose, Tmax - time of maximum concentration, $T_{1/2}$ - half life, FMO - flavin-containing monooxygenase) [96–99].

	Tigecycline	Omadacycline		Eravacycline	Sarecycline
**Dosage and route of administration**	initial dose of 100 mg iv; followed by iv infusion (30 minutes) of 50 mg every 12 hours	100 mg iv	300 mg orally	intermittent dosing-unique and repeated iv infusion (60 minutes) of 1 mg/kg every 12 hours	60 mg orally
**Pharmacokinetic parameters (medium values)**					
$C_{max\ ss}$ (μg/ml)	0.87	2.12	0.952	1.825	
AUC_{ss} (μg /L)	4.7 (AUC_{0-24h})	12.14	11.126	6.309 (AUC_{0-12h})	
Accumulation ratio		1.5		1.45	1.5-1.6
**Absorbtion**					
Bioavailability (%)	100	100	34.5	100	
Medium $T_{max\ ss}$ (hours)		0.5	2.5		
**Distribution**					
Plasma protein binding	71-89%	20% (independent of concentration)		79%-90% (increases with increasing plasma concentrations, with 79% to 90% at plasma concentrations ranging from 0.1 to 10 μg/mL)	62.5%-64.7%
Volume of distribution $_{ss}$ (L)	639	190	-	321	91.4-97

**Elimination**					
$T_{1/2\ ss}$	42.4	16	15.5	20	21-22
Systemic clearence $_{ss}$ (L/hour)	23.8	8.8	-		3 (apparent oral clearance)
Renal clearence $_{ss}$ (L/hour)	51.0	2,4-3,3			
**Metabolism**	<20%	No		CYP3A4 and FMO	<15% by microsomal enzymes

References

1. Nelson, M.L.; Levy, S.B. The History of the Tetracyclines. *Annals of the New York Academy of Sciences* **2011**, *1241*, 17–32, doi:10.1111/j.1749-6632.2011.06354.x.
2. PubChem Chlortetracycline Available online: https://pubchem.ncbi.nlm.nih.gov/compound/54675777 (accessed on 6 October 2021).
3. Finland, M. Twenty-Fifth Anniversary of the Discovery of Aureomycin: The Place of the Tetracyclines in Antimicrobial Therapy. *Clin Pharmacol Ther* **1974**, *15*, 3–8, doi:10.1002/cpt19741513.
4. Raistrick, H. Aureomycin, a New Antibiotic. *Nature* **1949**, *163*, 159–160, doi:10.1038/163159a0.
5. PubChem Oxytetracycline Available online: https://pubchem.ncbi.nlm.nih.gov/compound/54675779 (accessed on 6 October 2021).
6. PubChem Tetracycline Available online: https://pubchem.ncbi.nlm.nih.gov/compound/54675776 (accessed on 6 October 2021).
7. Stephens, C.R.; Conover, L.H.; Hochstein, F.A.; Regna, P.P.; Pilgrim, F.J.; Brunings, K.J.; Woodward, R.B. TERRAMYCIN. VIII. STRUCTURE OF AUREOMYCIN AND TERRAMYCIN. *J. Am. Chem. Soc.* **1952**, *74*, 4976–4977, doi:10.1021/ja01139a533.
8. PubChem Rolitetracycline Available online: https://pubchem.ncbi.nlm.nih.gov/compound/54682938 (accessed on 6 October 2021).
9. NCATS Inxight: Drugs — ROLITETRACYCLINE Available online: https://drugs.ncats.io/substance/GH9IW85221 (accessed on 6 October 2021).
10. PubChem Demeclocycline Available online: https://pubchem.ncbi.nlm.nih.gov/compound/54680690 (accessed on 6 October 2021).
11. PubChem Doxycycline Available online: https://pubchem.ncbi.nlm.nih.gov/compound/54671203 (accessed on 6 October 2021).
12. PubChem Minocycline Available online: https://pubchem.ncbi.nlm.nih.gov/compound/54675783 (accessed on 6 October 2021).
13. PubChem Methacycline Available online: https://pubchem.ncbi.nlm.nih.gov/compound/54675785 (accessed on 6 October 2021).
14. Lucelia, M. da S.; Herida, R.N.S. Methacycline: A Review of Analytical Methods. *Current Pharmaceutical Analysis* **2012**, *8*, 2–13.
15. PubChem Lymecycline Available online: https://pubchem.ncbi.nlm.nih.gov/compound/54707177 (accessed on 6 October 2021).
16. Tetralysal 300mg Hard Capsules - Summary of Product Characteristics (SmPC) - (Emc) Available online: https://www.medicines.org.uk/emc/product/926/smpc#gref (accessed on 29 September 2021).
17. Link: RCP Tygacil FDA Available online: https://www.accessdata.fda.gov/drugsatfda_docs/label/2007/021821s007lbl.pdf.
18. Tygacil | European Medicines Agency Available online: https://www.ema.europa.eu/en/medicines/human/EPAR/tygacil (accessed on 2 October 2021).
19. Stein, G.E.; Babinchak, T. Tigecycline: An Update. *Diagnostic Microbiology and Infectious Disease* **2013**, *75*, 331–336, doi:10.1016/j.diagmicrobio.2012.12.004.
20. Zhanel, G.G.; Esquivel, J.; Zelenitsky, S.; Lawrence, C.K.; Adam, H.J.; Golden, A.; Hink, R.; Berry, L.; Schweizer, F.; Zhanel, M.A.; et al. Omadacycline: A Novel Oral and Intravenous Aminomethylcycline Antibiotic Agent. *Drugs* **2020**, *80*, 285–313, doi:10.1007/s40265-020-01257-4.
21. Heaney, M.; Mahoney, M. V.; Gallagher, J.C. Eravacycline: The Tetracyclines Strike Back. *Annals of Pharmacotherapy* **2019**, *53*, 1124–1135, doi:10.1177/1060028019850173.

22. Alosaimy, S.; Abdul-Mutakabbir, J.C.; Kebriaei, R.; Jorgensen, S.C.J.; Rybak, M.J. Evaluation of Eravacycline: A Novel Fluorocycline. *Pharmacotherapy* **2020**, *40*, 221–238, doi:10.1002/phar.2366.
23. Haidari, W.; Bruinsma, R.; Cardenas-de la Garza, J.A.; Feldman, S.R. Sarecycline Review. *Annals of Pharmacotherapy* **2020**, *54*, 164–170, doi:10.1177/1060028019873111.
24. PubChem Tigecycline Available online: https://pubchem.ncbi.nlm.nih.gov/compound/54686904 (accessed on 2 August 2021).
25. Tigecycline---Chemical Information Search Available online: https://www.chemicalbook.com/ProductList_En.aspx?kwd=tigecycline (accessed on 5 October 2021).
26. Tigecycline | DrugBank Online Available online: https://go.drugbank.com/drugs/DB00560 (accessed on 2 August 2021).
27. Arias, K.; Robinson, S.G.; Lyngaas, S.S.; Cherala, S.S.; Hartzell, M.; Mei, S.; Vilic, A.; Girel, J.K.; Kuemmell, A.; Vrettos, J.S.; et al. Minocycline and Tigecycline Form Higher-Order Ca2+ Complexes of Stronger Affinity than Tetracycline. *Inorganica Chimica Acta* **2016**, *441*, 181–191, doi:10.1016/j.ica.2015.10.037.
28. 220620-09-7 Tigecycline AKSci E414 Available online: https://aksci.com/item_detail.php?cat=E414 (accessed on 5 October 2021).
29. Silva, L.M. da; Almeida, A.E. de; Salgado, H.R.N. Thermal Analysis and Validation of UV and Visible Spectrophotometric Methods for the Determination of New Antibiotic Tigecycline in Pharmaceutical Product. *Advances in Analytical Chemistry* **2012**, *2*, 10–15.
30. PubChem Omadacycline Available online: https://pubchem.ncbi.nlm.nih.gov/compound/54697325 (accessed on 5 October 2021).
31. Omadacycline Available online: https://go.drugbank.com/drugs/DB12455 (accessed on 7 October 2021).
32. Omadacycline | 389139-89-3 Available online: http://www.sigmaaldrich.com/ (accessed on 7 October 2021).
33. Omadacycline Tosylate | ≥99%(HPLC) | Selleck | Bacterial Chemical Available online: https://www.selleckchem.com/products/omadacycline-tosylate.html (accessed on 5 October 2021).
34. Omadacycline (Tosylate) Available online: https://www.chemsrc.com/en/cas/1075240-43-5_1349361.html (accessed on 5 October 2021).
35. Omadacycline Tosylate | DrugBank Online Available online: https://go.drugbank.com/salts/DBSALT002607 (accessed on 7 October 2021).
36. PubChem Eravacycline Available online: https://pubchem.ncbi.nlm.nih.gov/compound/54726192 (accessed on 7 October 2021).
37. European Medicines Agency Xerava Available online: https://www.ema.europa.eu/en/medicines/human/EPAR/xerava (accessed on 3 August 2021).
38. Eravacycline Available online: https://go.drugbank.com/drugs/DB12329 (accessed on 7 October 2021).
39. PubChem Eravacycline Dihydrochloride Available online: https://pubchem.ncbi.nlm.nih.gov/compound/56951485 (accessed on 7 October 2021).
40. Eravacycline Dihydrochloride | DrugBank Online Available online: https://go.drugbank.com/salts/DBSALT002787 (accessed on 7 October 2021).
41. Eravacycline Dihydrochloride (TP-434 Dihydrochloride) | Anti-Bacterial Agent | MedChemExpress Available online: https://www.medchemexpress.com/Eravacycline_dihydrochloride.html (accessed on 7 October 2021).
42. PubChem Sarecycline Available online: https://pubchem.ncbi.nlm.nih.gov/compound/54681908 (accessed on 4 October 2021).
43. Sarecycline Available online: https://go.drugbank.com/drugs/DB12035 (accessed on 7 October 2021).
44. Welcome to Medkoo Biosciences Available online: https://medkoo.com/ (accessed on 7 October 2021).
45. Sarecycline Hydrochloride | C24H30ClN3O8 - PubChem Available online: https://pubchem.ncbi.nlm.nih.gov/compound/71296095 (accessed on 7 October 2021).
46. Sarecycline Hydrochloride | DrugBank Online Available online: https://go.drugbank.com/salts/DBSALT002125 (accessed on 7 October 2021).
47. Sarecycline Hydrochloride | Antibiotic | MedChemExpress Available online: https://www.medchemexpress.com/sarecycline-hydrochloride.html (accessed on 7 October 2021).
48. Noskin, G.A. Tigecycline: A New Glycylcycline for Treatment of Serious Infections. *Clinical Infectious Diseases* **2005**, *41*, S303–S314, doi:10.1086/431672.

49. Biedenbach, D.J.; Beach, M.L.; Jones, R.N. In Vitro Antimicrobial Activity of GAR-936 Tested against Antibiotic-Resistant Gram-Positive Blood Stream Infection Isolates and Strains Producing Extended-Spectrum β-Lactamases. *Diagnostic Microbiology and Infectious Disease* **2001**, *40*, 173–177, doi:10.1016/S0732-8893(01)00269-3.
50. Postier, R.G.; Green, S.L.; Klein, S.R.; Ellis-Grosse, E.J.; Loh, E.; Tigecycline 200 Study Group Results of a Multicenter, Randomized, Open-Label Efficacy and Safety Study of Two Doses of Tigecycline for Complicated Skin and Skin-Structure Infections in Hospitalized Patients. *Clin Ther* **2004**, *26*, 704–714, doi:10.1016/s0149-2918(04)90070-7.
51. Petersen, P.J.; Jacobus, N.V.; Weiss, W.J.; Sum, P.E.; Testa, R.T. In Vitro and in Vivo Antibacterial Activities of a Novel Glycylcycline, the 9-t-Butylglycylamido Derivative of Minocycline (GAR-936). *Antimicrob Agents Chemother* **1999**, *43*, 738–744, doi:10.1128/AAC.43.4.738.
52. van Ogtrop, M.L.; Andes, D.; Stamstad, T.J.; Conklin, B.; Weiss, W.J.; Craig, W.A.; Vesga, O. In Vivo Pharmacodynamic Activities of Two Glycylcyclines (GAR-936 and WAY 152,288) against Various Gram-Positive and Gram-Negative Bacteria. *Antimicrob Agents Chemother* **2000**, *44*, 943–949, doi:10.1128/AAC.44.4.943-949.2000.
53. Betriu, C.; Rodríguez-Avial, I.; Sánchez, B.A.; Gómez, M.; Álvarez, J.; Picazo, J.J. In Vitro Activities of Tigecycline (GAR-936) against Recently Isolated Clinical Bacteria in Spain. *Antimicrob Agents Chemother* **2002**, *46*, 892–895, doi:10.1128/AAC.46.3.892-895.2002.
54. Goldstein, E.J.; Citron, D.M.; Merriam, C.V.; Warren, Y.; Tyrrell, K. Comparative in Vitro Activities of GAR-936 against Aerobic and Anaerobic Animal and Human Bite Wound Pathogens. *Antimicrob Agents Chemother* **2000**, *44*, 2747–2751, doi:10.1128/AAC.44.10.2747-2751.2000.
55. Milatovic, D.; Schmitz, F.-J.; Verhoef, J.; Fluit, A.C. Activities of the Glycylcycline Tigecycline (GAR-936) against 1,924 Recent European Clinical Bacterial Isolates. *Antimicrob Agents Chemother* **2003**, *47*, 400–404, doi:10.1128/AAC.47.1.400-404.2003.
56. Cercenado, E.; Cercenado, S.; Gómez, J.A.; Bouza, E. In Vitro Activity of Tigecycline (GAR-936), a Novel Glycylcycline, against Vancomycin-Resistant Enterococci and Staphylococci with Diminished Susceptibility to Glycopeptides. *J Antimicrob Chemother* **2003**, *52*, 138–139, doi:10.1093/jac/dkg289.
57. Low, D.E.; Kreiswirth, B.N.; Weiss, K.; Willey, B.M. Activity of GAR-936 and Other Antimicrobial Agents against North American Isolates of Staphylococcus Aureus. *Int J Antimicrob Agents* **2002**, *20*, 220–222, doi:10.1016/s0924-8579(02)00132-2.
58. Boucher, H.W.; Wennersten, C.B.; Eliopoulos, G.M. In Vitro Activities of the Glycylcycline GAR-936 against Gram-Positive Bacteria. *Antimicrobial Agents and Chemotherapy* **2000**, *44*, 2225–2229, doi:10.1128/AAC.44.8.2225-2229.2000.
59. Kitzis, M.D.; Ly, A.; Goldstein, F.W. In Vitro Activities of Tigecycline (GAR-936) against Multidrug-Resistant Staphylococcus Aureus and Streptococcus Pneumoniae. *Antimicrob Agents Chemother* **2004**, *48*, 366–367, doi:10.1128/AAC.48.1.366-367.2004.
60. Zhanel, G.G.; Palatnick, L.; Nichol, K.A.; Bellyou, T.; Low, D.E.; Hoban, D.J. Antimicrobial Resistance in Respiratory Tract Streptococcus Pneumoniae Isolates: Results of the Canadian Respiratory Organism Susceptibility Study, 1997 to 2002. *Antimicrobial Agents and Chemotherapy* **2003**, *47*, 1867–1874, doi:10.1128/AAC.47.6.1867-1874.2003.
61. Betriu, C.; Culebras, E.; Rodríguez-Avial, I.; Gómez, M.; Sánchez, B.A.; Picazo, J.J. In Vitro Activities of Tigecycline against Erythromycin-Resistant Streptococcus Pyogenes and Streptococcus Agalactiae: Mechanisms of Macrolide and Tetracycline Resistance. *Antimicrob Agents Chemother* **2004**, *48*, 323–325, doi:10.1128/AAC.48.1.323-325.2004.
62. Patel, R.; Rouse, M.S.; Piper, K.E.; Steckelberg, J.M. In Vitro Activity of GAR-936 against Vancomycin-Resistant Enterococci, Methicillin-Resistant Staphylococcus Aureus and Penicillin-Resistant Streptococcus Pneumoniae. *Diagn Microbiol Infect Dis* **2000**, *38*, 177–179, doi:10.1016/s0732-8893(00)00193-0.
63. Petersen, P.J.; Bradford, P.A.; Weiss, W.J.; Murphy, T.M.; Sum, P.E.; Projan, S.J. In Vitro and in Vivo Activities of Tigecycline (GAR-936), Daptomycin, and Comparative Antimicrobial Agents against Glycopeptide-Intermediate Staphylococcus Aureus and Other Resistant Gram-Positive Pathogens. *Antimicrob Agents Chemother* **2002**, *46*, 2595–2601, doi:10.1128/AAC.46.8.2595-2601.2002.

64. Gales, A.C.; Jones, R.N.; Andrade, S.S.; Pereira, A.S.; Sader, H.S. In Vitro Activity of Tigecycline, a New Glycylcycline, Tested against 1,326 Clinical Bacterial Strains Isolated from Latin America. *Braz J Infect Dis* **2005**, *9*, 348–356, doi:10.1590/s1413-86702005000500001.
65. Betriu, C.; Rodríguez-Avial, I.; Sánchez, B.A.; Gómez, M.; Picazo, J.J. Comparative in Vitro Activities of Tigecycline (GAR-936) and Other Antimicrobial Agents against Stenotrophomonas Maltophilia. *J Antimicrob Chemother* **2002**, *50*, 758–759, doi:10.1093/jac/dkf196.
66. Henwood, C.J.; Gatward, T.; Warner, M.; James, D.; Stockdale, M.W.; Spence, R.P.; Towner, K.J.; Livermore, D.M.; Woodford, N. Antibiotic Resistance among Clinical Isolates of Acinetobacter in the UK, and in Vitro Evaluation of Tigecycline (GAR-936). *J Antimicrob Chemother* **2002**, *49*, 479–487, doi:10.1093/jac/49.3.479.
67. Edlund, C.; Nord, C.E. In-Vitro Susceptibility of Anaerobic Bacteria to GAR-936, a New Glycylcycline. *Clin Microbiol Infect* **2000**, *6*, 159–163, doi:10.1046/j.1469-0691.2000.00034-6.x.
68. Wallace, R.J.; Brown-Elliott, B.A.; Crist, C.J.; Mann, L.; Wilson, R.W. Comparison of the in Vitro Activity of the Glycylcycline Tigecycline (Formerly GAR-936) with Those of Tetracycline, Minocycline, and Doxycycline against Isolates of Nontuberculous Mycobacteria. *Antimicrob Agents Chemother* **2002**, *46*, 3164–3167, doi:10.1128/AAC.46.10.3164-3167.2002.
69. Rhomberg, P.R.; Jones, R.N. In Vitro Activity of 11 Antimicrobial Agents, Including Gatifloxacin and GAR936, Tested against Clinical Isolates of Mycobacterium Marinum. *Diagn Microbiol Infect Dis* **2002**, *42*, 145–147, doi:10.1016/s0732-8893(01)00332-7.
70. Roblin, P.M.; Hammerschlag, M.R. In Vitro Activity of GAR-936 against Chlamydia Pneumoniae and Chlamydia Trachomatis. *Int J Antimicrob Agents* **2000**, *16*, 61–63, doi:10.1016/s0924-8579(00)00198-9.
71. Grossman, T.H. Tetracycline Antibiotics and Resistance. *Cold Spring Harb Perspect Med* **2016**, *6*, a025387, doi:10.1101/cshperspect.a025387.
72. Zhanel, G.G.; Esquivel, J.; Zelenitsky, S.; Lawrence, C.K.; Adam, H.J.; Golden, A.; Hink, R.; Berry, L.; Schweizer, F.; Zhanel, M.A.; et al. Omadacycline: A Novel Oral and Intravenous Aminomethylcycline Antibiotic Agent. *Drugs* **2020**, *80*, 285–313, doi:10.1007/s40265-020-01257-4.
73. Zhanel, G.G.; Homenuik, K.; Nichol, K.; Noreddin, A.; Vercaigne, L.; Embil, J.; Gin, A.; Karlowsky, J.A.; Hoban, D.J. The Glycylcyclines: A Comparative Review with the Tetracyclines. *Drugs* **2004**, *64*, 63–88, doi:10.2165/00003495-200464010-00005.
74. Karlowsky, J.A.; Steenbergen, J.; Zhanel, G.G. Microbiology and Preclinical Review of Omadacycline. *Clin Infect Dis* **2019**, *69*, S6–S15, doi:10.1093/cid/ciz395.
75. Huband, M.D.; Pfaller, M.A.; Shortridge, D.; Flamm, R.K. Surveillance of Omadacycline Activity Tested against Clinical Isolates from the United States and Europe: Results from the SENTRY Antimicrobial Surveillance Programme, 2017. *J Glob Antimicrob Resist* **2019**, *19*, 56–63, doi:10.1016/j.jgar.2019.02.017.
76. Pfaller, M.A.; Rhomberg, P.R.; Huband, M.D.; Flamm, R.K. Activity of Omadacycline Tested against Streptococcus Pneumoniae from a Global Surveillance Program (2014). *Diagn Microbiol Infect Dis* **2018**, *90*, 143–147, doi:10.1016/j.diagmicrobio.2017.10.010.
77. Pfaller, M.A.; Rhomberg, P.R.; Huband, M.D.; Flamm, R.K. Activities of Omadacycline and Comparator Agents against Staphylococcus Aureus Isolates from a Surveillance Program Conducted in North America and Europe. *Antimicrob Agents Chemother* **2017**, *61*, e02411-16, doi:10.1128/AAC.02411-16.
78. Pfaller, M.A.; Huband, M.D.; Rhomberg, P.R.; Flamm, R.K. Surveillance of Omadacycline Activity against Clinical Isolates from a Global Collection (North America, Europe, Latin America, Asia-Western Pacific), 2010-2011. *Antimicrob Agents Chemother* **2017**, *61*, e00018-17, doi:10.1128/AAC.00018-17.
79. Lee, H.; Kim, H.; Seo, Y.H.; Yong, D.; Jeong, S.H.; Lee, K.; Chong, Y. In Vitro Activity of Tigecycline Alone and Antimicrobial Combinations against Clinical Neisseria Gonorrhoeae Isolates. *Diagn Microbiol Infect Dis* **2017**, *87*, 160–162, doi:10.1016/j.diagmicrobio.2016.10.022.
80. Stapert, L.; Wolfe, C.; Shinabarger, D.; Marra, A.; Pillar, C. In Vitro Activities of Omadacycline and Comparators against Anaerobic Bacteria. *Antimicrob Agents Chemother* **2018**, *62*, e00047-18, doi:10.1128/AAC.00047-18.
81. Shoen, C.; Benaroch, D.; Sklaney, M.; Cynamon, M. In Vitro Activities of Omadacycline against Rapidly Growing Mycobacteria. *Antimicrobial Agents and Chemotherapy* **2019**, *63*, e02522-18, doi:10.1128/AAC.02522-18.

82. Goldstein, E.J.C.; Citron, D.M.; Tyrrell, K.L.; Leoncio, E.; Merriam, C.V. Comparative In Vitro Activity of Omadacycline against Dog and Cat Bite Wound Isolates. *Antimicrob Agents Chemother* **2018**, *62*, e02551-17, doi:10.1128/AAC.02551-17.
83. Steenbergen, J.; Tanaka, S.K.; Miller, L.L.; Halasohoris, S.A.; Hershfield, J.R. In Vitro and In Vivo Activity of Omadacycline against Two Biothreat Pathogens, Bacillus Anthracis and Yersinia Pestis. *Antimicrob Agents Chemother* **2017**, *61*, e02434-16, doi:10.1128/AAC.02434-16.
84. Pfaller, M.A.; Rhomberg, P.R.; Huband, M.D.; Flamm, R.K. Activity of Omadacycline Tested against Enterobacteriaceae Causing Urinary Tract Infections from a Global Surveillance Program (2014). *Diagn Microbiol Infect Dis* **2018**, *91*, 179–183, doi:10.1016/j.diagmicrobio.2018.01.019.
85. Carvalhaes, C.G.; Huband, M.D.; Reinhart, H.H.; Flamm, R.K.; Sader, H.S. Antimicrobial Activity of Omadacycline Tested against Clinical Bacterial Isolates from Hospitals in Mainland China, Hong Kong, and Taiwan: Results from the SENTRY Antimicrobial Surveillance Program (2013 to 2016). *Antimicrob Agents Chemother* **2019**, *63*, e02262-18, doi:10.1128/AAC.02262-18.
86. Fluit, A.C.; van Gorkum, S.; Vlooswijk, J. Minimal Inhibitory Concentration of Omadacycline and Doxycycline against Bacterial Isolates with Known Tetracycline Resistance Determinants. *Diagn Microbiol Infect Dis* **2019**, *94*, 78–80, doi:10.1016/j.diagmicrobio.2018.11.010.
87. Waites, K.B.; Crabb, D.M.; Liu, Y.; Duffy, L.B. In Vitro Activities of Omadacycline (PTK 0796) and Other Antimicrobial Agents against Human Mycoplasmas and Ureaplasmas. *Antimicrob Agents Chemother* **2016**, *60*, 7502–7504, doi:10.1128/AAC.01734-16.
88. Kohlhoff, S.A.; Huerta, N.; Hammerschlag, M.R. In Vitro Activity of Omadacycline against Chlamydia Pneumoniae. *Antimicrob Agents Chemother* **2019**, *63*, e01907-18, doi:10.1128/AAC.01907-18.
89. Sutcliffe, J.A.; O'Brien, W.; Fyfe, C.; Grossman, T.H. Antibacterial Activity of Eravacycline (TP-434), a Novel Fluorocycline, against Hospital and Community Pathogens. *Antimicrob. Agents Chemother.* **2013**, *57*, 5548–5558, doi:10.1128/AAC.01288-13.
90. Solomkin, J.S.; Ramesh, M.K.; Cesnauskas, G.; Novikovs, N.; Stefanova, P.; Sutcliffe, J.A.; Walpole, S.M.; Horn, P.T. Phase 2, Randomized, Double-Blind Study of the Efficacy and Safety of Two Dose Regimens of Eravacycline versus Ertapenem for Adult Community-Acquired Complicated Intra-Abdominal Infections. *Antimicrob Agents Chemother* **2014**, *58*, 1847–1854, doi:10.1128/AAC.01614-13.
91. Abdallah, M.; Olafisoye, O.; Cortes, C.; Urban, C.; Landman, D.; Quale, J. Activity of Eravacycline against Enterobacteriaceae and Acinetobacter Baumannii, Including Multidrug-Resistant Isolates, from New York City. *Antimicrob Agents Chemother* **2015**, *59*, 1802–1805, doi:10.1128/AAC.04809-14.
92. Zhanel, G.G.; Baxter, M.R.; Adam, H.J.; Sutcliffe, J.; Karlowsky, J.A. In Vitro Activity of Eravacycline against 2213 Gram-Negative and 2424 Gram-Positive Bacterial Pathogens Isolated in Canadian Hospital Laboratories: CANWARD Surveillance Study 2014-2015. *Diagn Microbiol Infect Dis* **2018**, *91*, 55–62, doi:10.1016/j.diagmicrobio.2017.12.013.
93. Snydman, D.R.; McDermott, L.A.; Jacobus, N.V.; Kerstein, K.; Grossman, T.H.; Sutcliffe, J.A. Evaluation of the In Vitro Activity of Eravacycline against a Broad Spectrum of Recent Clinical Anaerobic Isolates. *Antimicrobial Agents and Chemotherapy* **2018**, *62*, e02206-17, doi:10.1128/AAC.02206-17.
94. Zhanel, G.; Critchley, I.; Lin, L.-Y.; Alvandi, N. Microbiological Profile of Sarecycline, a Novel Targeted Spectrum Tetracycline for the Treatment of Acne Vulgaris. *Antimicrob. Agents Chemother.* **2019**, *63*, e01297-18, doi:10.1128/AAC.01297-18.
95. Moore, A.Y.; Charles, J.E.M.; Moore, S. Sarecycline: A Narrow Spectrum Tetracycline for the Treatment of Moderate-to-Severe Acne Vulgaris. *Future Microbiol.* **2019**, *14*, 1235–1242, doi:10.2217/fmb-2019-0199.
96. FDA Drug Approval Package: NUZYRA Available online: https://www.accessdata.fda.gov/drugsatfda_docs/nda/2018/209816Orig1s000,209817Orig1s000TOC.cfm (accessed on 4 October 2021).
97. FDA Drug Approval Package: Seysara (Sarecycline) Available online: https://www.accessdata.fda.gov/drugsatfda_docs/nda/2018/209521Orig1s000TOC.cfm (accessed on 7 October 2021).

98. FDA Drug Approval Package: Tygacil (Tigecycline) NDA #021821 Available online: https://www.accessdata.fda.gov/drugsatfda_docs/nda/2005/21-821_Tygacil.cfm (accessed on 4 October 2021).
99. FDA Drug Approval Package: XERAVA (Eravacycline) Available online: https://www.accessdata.fda.gov/drugsatfda_docs/nda/2018/211109Orig1s000TOC.cfm (accessed on 30 November 2021).
